# Reductive photoredox transformations of carbonyl derivatives enabled by strongly reducing photosensitizers†

**DOI:** 10.1039/d3sc03000h

**Published:** 2023-08-18

**Authors:** Vinh Q. Dang, Thomas S. Teets

**Affiliations:** a University of Houston, Department of Chemistry 3585 Cullen Blvd. Room 112 Houston TX 77204-5003 USA tteets@uh.edu

## Abstract

Visible-light photoredox catalysis is well-established as a powerful and versatile organic synthesis strategy. However, some substrate classes, despite being attractive precursors, are recalcitrant to single-electron redox chemistry and thus not very amenable to photoredox approaches. Among these are carbonyl derivatives, *e.g.* ketones, aldehydes, and imines, which in most cases require Lewis or Brønsted acidic additives to activate *via* photoinduced electron transfer. In this work, we unveil a range of photoredox transformations on ketones and imines, enabled by strongly reducing photosensitizers and operating under simple, general conditions with a single sacrificial reductant and no additives. Specific reactions described here are umpolung C–C bond forming reactions between aromatic ketones or imines and electron-poor alkenes, imino-pinacol homocoupling reactions of challenging alkyl-aryl imine substrates, and γ-lactonization reactions of aromatic ketones with methyl acrylate. The reactions are all initiated by photoinduced electron transfer to form a ketyl or iminyl that is subsequently trapped.

## Abstract 2

A strongly photoreducing iridium photosensitizer enables diverse, additive-free reductive photoredox transformations on challenging ketone and imine substrates.

## Graphical abstract

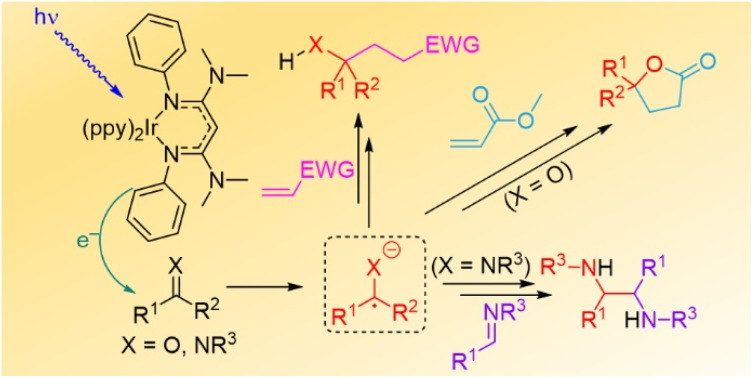

## Introduction

Photoredox catalysis has become a mainstream, versatile strategy for small-molecule^1^ and polymer^2^ organic synthesis applications.^3^ Most organic photoredox reactions involve the generation of radicals by photoinduced electron transfer. A wide range of outcomes for these photogenerated radicals are possible, which depend on the nature of the radical and other reaction partners that are present.^4^ A variety of substrates can be used as radical precursors in photoredox catalysis; much early work focused on activated organohalides, sulfones, and sulfoniums, which are comparatively easy to reduce,^5–7^ whereas more recently unactivated organohalide substrates, much more challenging to reduce, have emerged as viable options.^8–15^ The efficient generation of radicals in a photoredox transformation depends on the thermodynamics and kinetics of photoinduced electron transfer reactions, which are determined by the excited-state redox properties of the photosensitizer, often referred to as the photocatalyst.^16^ As such, the choice of photosensitizer used in a photoredox transformation is critical.

Ketones, imines, and related carbonyl-derived substrate classes are attractive for photoredox catalysis. Unlike many of the above-mentioned radical precursors, *e.g.*, organohalides, reduction of carbonyl substrates to radicals is not accompanied by loss of a leaving group, which improves atom economy. Furthermore, since the heteroatom functional group from the substrate remains in the product, these carbonyl derivatives can be used as precursors for alcohols, amines, and heterocyclic products.^17^ Traditional methods for reductive transformations of ketones and imines require strong reducing agents and harsh reaction conditions to form the corresponding ketyl or iminyl.^17,18^ (Note: The terms “ketyl” and “iminyl” specifically refer to the respective radical anions, formed by one-electron reduction of the ketone or imine substrate). Photoredox catalysis has enabled these reactive radicals to be generated more mildly. A very common outcome in photoredox transformations of ketones and imines is dimerization of the respective radical to form pinacol or 1,2-diamine products.^19–27^ There are several advances that have allowed photogenerated ketyl intermediates to engage in more synthetically useful heterocoupling and cyclization reactions.^17,28^ Intramolecular approaches, where the ketyl is tethered to an alkene or alkyne functional group, have been effective for making cyclic products.^29–31^ Strategies have also been developed to overcome the difficulties of reducing ketone and imine substrates to generate the key radical intermediate. Some works use photosensitizers that absorb high-energy violet or UV light,^29,30,32^ which are reactive enough to generate ketyl intermediates even when aliphatic ketone or aldehyde substrates are used, a category of substrates not yet accessible with visible-light irradiation. Another common approach uses Lewis acid^33^ additives that can bind to the substrate or other additives like Hantzsch ester that can function as Brønsted acids, protonating the substrate or enabling PCET elementary steps.^34–37^ These strategies all serve to make the substrate easier to reduce and facilitate the one-electron reduction step that generates the ketyl or iminyl intermediate, but they come at the expense of atom economy.

**Fig. 1:**
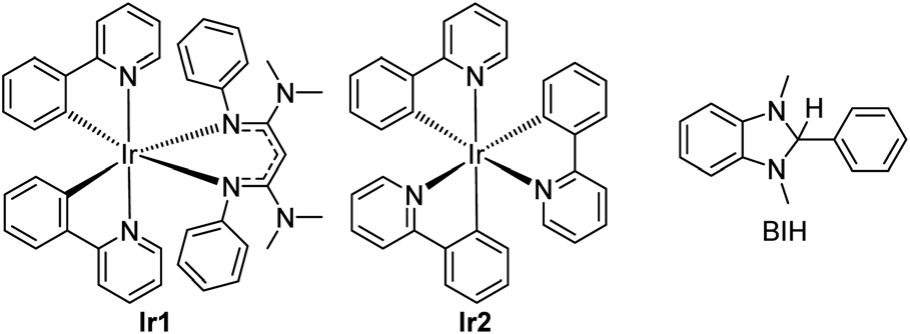
Structures of photosensitizers (Ir1 and Ir2) and sacrificial reductant (BIH) used in this study.

In short, whereas the above-mentioned advances have allowed diverse and synthetically useful photoredox transformations to be executed on carbonyl derivatives, they all require judicious substrate design, the inclusion of additives, and/or the use of short-wavelength photosensitizers. In this work, we show that strongly reducing photosensitizers recently developed by our group enable a diverse range of transformations on ketones and imines under simple, generalizable visible-light conditions, overcoming some of the key challenges with these substrate classes. Chief among these, ketones and imines are difficult to reduce, typically with reduction potentials that lie well beyond −2.0 V *vs.* the ferrocenium/ferrocene (Fc^+^/Fc) couple (−1.6 V *vs.* SCE).^38,39^ Their redox-inert nature is the main reason why UV photosensitizers or acidic additives are often needed, and motivates the search for highly reducing photosensitizers and/or alternative reaction conditions that can efficiently generate and functionalize carbonyl substrates. Along these lines, our group has developed a class of potent visible-light photoreductants of the general formula Ir(ppy)_2_(NacNac^R^), where ppy = 2-phenylpyridine and NacNac^R^ is a substituted β-diketiminate.^14,40^ One such member of this series, Ir(ppy)_2_(NacNac^NMe2^) (Ir1, see Fig. 1) has an excited-state potential *E*(Ir^IV^/*Ir^III^) of −2.6 V *vs.* Fc^+^/Fc, more potent by 500 mV when compared to *fac*-Ir(ppy)_3_ (Ir2), commonly used as a photocatalyst for challenging reductive transformations. Ir1 is capable of catalytically photoreducing a variety of inert organohalide substrates under visible-light irradiation.^14,15^ Relevant to the current work, we have also shown, in stoichiometric Stern–Volmer quenching studies, that Ir1 rapidly transfers electrons to benzophenone or acetophenone upon excitation,^41^ and an isolated catalytic screen with benzophenone, Ir1, and the sacrificial reductant 1,3-dimethyl-2,3-dihydro-2-phenyl-benzimidazole (BIH, see Fig. 1) under blue light yielded quantitative hydrogenation to diphenylmethanol.^15^ This catalytic outcome stands in contrast to the normally observed pinacol dimerization under similar conditions.^19^

Both the photocatalytic outcome and the stoichiometric electron transfer studies referenced above suggest that Ir1 is particularly adept at generating ketyls. This motivated us to investigate Ir1 as a photocatalyst for synthetically useful photoredox reactions of benzophenone, and to extend to other related carbonyl substrate classes. In this work, we show that under very simple and generalizable conditions, Ir1 promotes a variety of radical-based functionalization reactions of ketones and imines. These include umpolung C–C coupling reactions using Michael acceptor coupling partners, imino-pinacol coupling reactions of recalcitrant imine substrates, and γ-lactonization reactions between ketone substrates and acrylate coupling partners. Importantly, all these transformations proceed using only a single sacrificial reductant (BIH) with no other additives and demonstrate that strongly reducing photosensitizers can be critical components in developing wide-ranging transformations of ketone and imine substrates.

## Results and discussion

### Umpolung C–C bond formation of ketones (Michael addition)

The experimental setup for photoredox reactions is documented in Fig. S1 of the ESI,† using commercial blue LEDs with a spectral profile shown in Fig. S2† that overlaps well with the UV-vis absorption spectrum of Ir1, Fig. S3.† Leveraging the strong reducing ability of Ir1 and its ability to efficiently generate ketyls under visible-light irradiation,^15,40^ we initially targeted umpolung C–C bond forming reactions where the photogenerated ketyl is trapped by electron-deficient alkenes in what can be formalized as a Michael addition reaction.^42^ To optimize reaction conditions, the coupling of benzophenone with phenyl vinyl sulfone was first studied. Table 1 summarizes these outcomes, quoting yields determined by gas chromatography (GC). Reactions employing common trialkylamine sacrificial reductants, often used in reductive dimerization reactions of carbonyl substrates,^19,23^ yielded no desired product with recovery of unreacted benzophenone (entries 1–4). We attribute these outcomes to the poor redox potential matching of amines with the Ir^IV^/Ir^III^ couple of the photosensitizer. However, switching to BIH as the sacrificial reagent produced near-quantitative yield (95%) of the coupled alcohol product (entry 5). Importantly, high yields of the alcohol product are obtained without any Lewis or Brønsted acidic additives. As shown in Fig. S4 of the ESI,† a full 10 equivalents of phenyl vinyl sulfone are required for complete conversion. We next screened a variety of other polar organic solvents (entries 5–9) and obtained the best outcome with DMF, with DMSO (87%) also proving satisfactory. We also found that reducing the catalyst loading from 5 mol% to 1 mol% had minimal impact on reaction yield (entry 10), and that Ir2, *fac*-Ir(ppy)_3_, is also capable of promoting this transformation with only slightly diminished yield (entry 11), suggesting that any photosensitizer reducing enough to generate the ketyl could work under these conditions. Finally, in a series of control reactions (entries 12 and 13) we showed that all reaction components are necessary; omitting the photosensitizer (entry 12) gave no conversion of benzophenone with dimerized phenyl vinyl sulfone as the only detected product and omitting the light source (entry 13) resulted in no conversion at all. Entries 14 and 15 provide some mechanistic hints. Carrying out the reaction in air instead of inert atmosphere (entry 14) likewise results in no product yield, suggesting that the Ir1 triplet excited state and/or a photogenerated radical are quenched by O_2_, and including TEMPO as a radical trap (entry 15) likewise shuts down the reaction, indicating the involvement of radical intermediates.

**Table 1:** Optimization and control experiments for umpolung C–C coupling

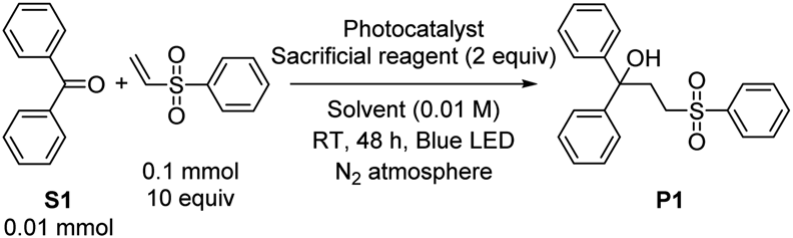				
Entry	Catalyst (mol%)	Sacrificial reagent^a^	Solvent	% Yield^b^
1	Ir1 (5%)	TMEDA	DMF	0
2	Ir1 (5%)	TBA	DMF	0
3	Ir1 (5%)	DIPEA	DMF	Trace
4	Ir1 (5%)	TEA	DMF	0
5	Ir1 (5%)	BIH	DMF	95
6	Ir1 (5%)	BIH	CH_3_CN	36
7	Ir1 (5%)	BIH	DMSO	87
8	Ir1 (5%)	BIH	THF	0
9	Ir1 (5%)	BIH	Et_2_O	Trace
10	Ir1 (1%)	BIH	DMF	93
11	Ir2 (1%)	BIH	DMF	87
12	None	BIH	DMF	0
13^c^	Ir1 (1%)	BIH	DMF	0
14^d^	Ir1 (1%)	BIH	DMF	0
15^e^	Ir1 (1%)	BIH	DMF	0

a TMEDA = tetramethylethylenediamine; TBA = tributylamine; DIPEA = *N*,*N*-diisopropylethylamine; TEA = triethylamine; BIH = 1,3-dimethyl-2,3-dihydro-2-phenyl-benzimidazole.

b Yield was determined by gas chromatography with 1,3,5-trimethoxybenzene as the internal standard.

c In dark.

d In air.

e With 2 equiv. of TEMPO added.

**Scheme 1:**
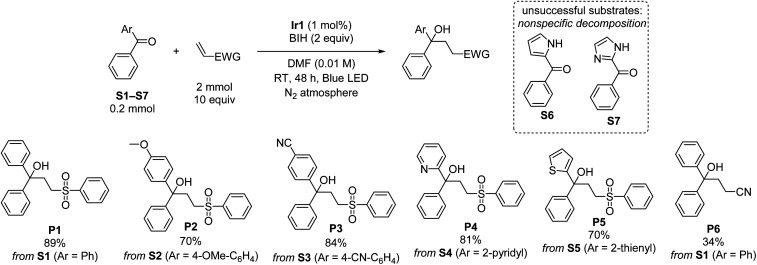
Substrate scope for ketone reductive coupling reactions, showing isolated yields.

Using the optimized conditions from entry 10 of Table 1, the scope of the reaction with respect to diarylketone and alkene was explored (Scheme 1). Note that in Scheme 1 and all subsequent reaction schemes isolated product yields are quoted; we estimate in most cases crude yields that are *ca.* 5–15% higher, with some product loss during purification. The reaction tolerates both electron-donating (S2) and electron-withdrawing (S3) groups on the benzophenone substrate, the latter giving slightly higher isolated yields when coupled with phenyl vinyl sulfone, likely a result of being easier to reduce. The reaction also works well with select heterocycles, with 81% yield of the coupled product for 2-benzoylpyridine (S4), and a slightly lower 70% yield for phenyl(thiophen-2-yl)methanone (S5). Pyrrole (S6) or imidazole (S7) derived substrates with acidic N–H protons are not amenable to this transformation. With the representative substrate S1 we also investigated alternative electron-poor alkene coupling partners, obtaining a modest product yield when acrylonitrile is substituted for phenyl vinyl sulfone (P6), but nonetheless suggesting that other Michael-acceptor alkenes could be employed in this reaction.

### Intermolecular imine Michael addition reactions

**Scheme 2:**
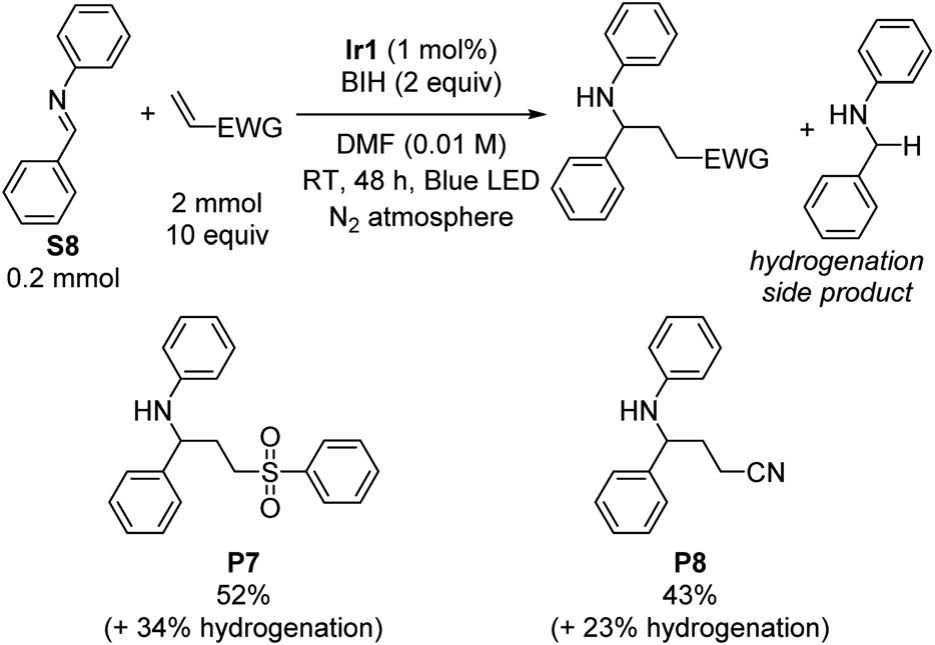
Imine reductive coupling reactions. Isolated yields are shown.

Having satisfactorily demonstrated photoreductive coupling reactions of ketones, we moved on to imine substrates, which are even more difficult to reduce on the basis of their highly negative reduction potentials.^38^ Using the same optimized conditions for reductive coupling of ketones with electron-poor alkenes, cross-coupling reactions of *N*-benzylideneaniline (S8) with phenyl vinyl sulfone and acrylonitrile were achieved (Scheme 2). Yields were slightly lower than typically observed with diaryl ketone substrates (52% for P7 and 43% for P8). As before, phenyl vinyl sulfone was a better radical trap than acrylonitrile, but for this transformation the difference was not as pronounced. There was no evidence for the formation of iminyl dimerization products, though we did observe the hydrogenation product *N*-benzylaniline (34% and 23% yield by GC in the case of phenyl vinyl sulfone and acrylonitrile, respectively) as a deleterious side product that limited the cross-coupling yield.

### Imino-pinacol coupling of challenging imines

**Scheme 3:**
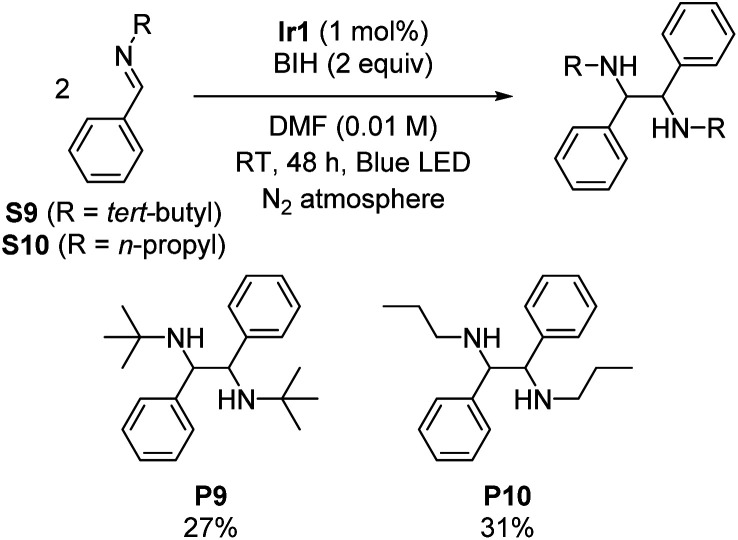
Imino-pinacol coupling reactions, showing isolated yields.

Symmetric diamines, generated through the reductive dimerization of imines, have garnered significant attention in the field of organic chemistry.^19^ However, previous studies mainly used imines with protecting groups or functional groups that engender milder reduction potentials.^19–22,27^ To push the boundaries of photoreductive coupling reactions of imimes, we capitalized on our strongly reducing photosensitizer to investigate challenging imines with very negative reduction potentials. Initially, we synthesized two *N*-alkyl imines, namely *N-tert*-butyl-1-phenylmethanimine (S9) and phenyl-*N*-propylmethanimine (S10). Cyclic voltammetry experiments confirm their highly negative reduction potentials, which show irreversible reduction waves with cathodic peak potentials of −2.96 V and −2.84 V *vs.* Fc^+^/Fc for S9 and S10, respectively (Fig. S5 and S6†). Scheme 3 summarizes the outcomes, showing successful dimerization of these substrates (27% yield for P9 and 31% yield for P10), albeit with modest yields due to their extreme reduction potentials. This result further demonstrates that the choice of photosensitizer can enable transformations of challenging substrates, showcasing the potential of this approach.

### Gamma-lactonization

**Scheme 4:**
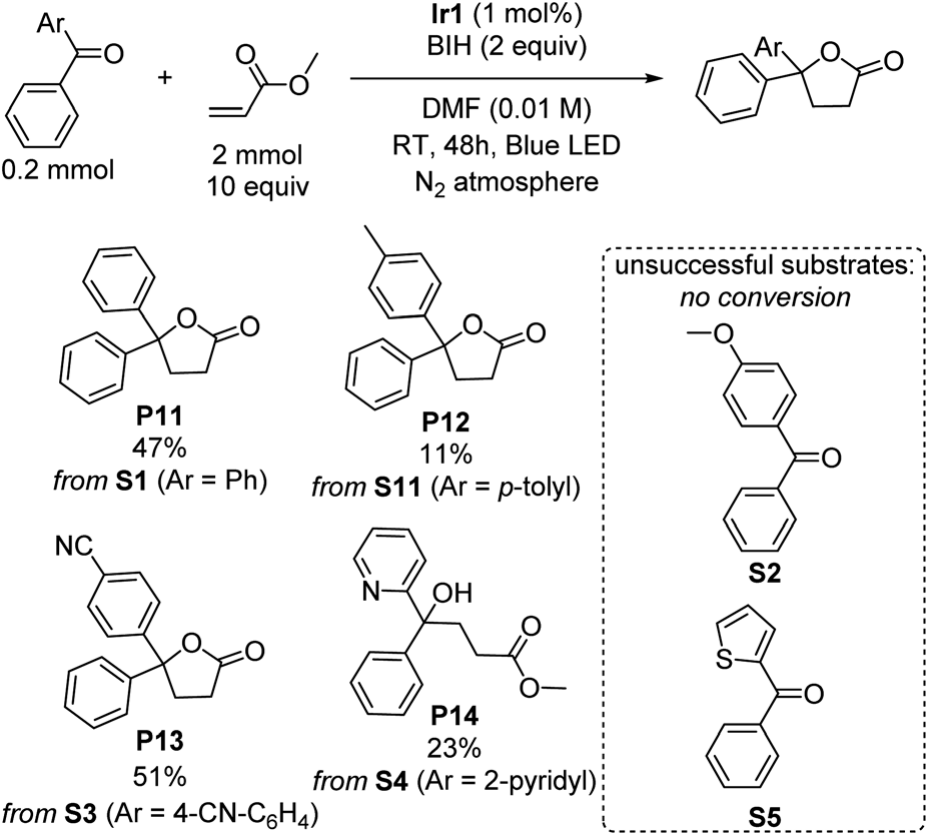
Lactonization of benzophenone derivatives. Isolated yields are indicated.

γ-Lactones are ubiquitous structural elements in natural products, pharmaceuticals, foods, and perfumes.^43^ Therefore, various approaches have been developed to incorporate lactones into organic compounds. Traditional methods include intra- or intermolecular reactions of alkenes, alcohols, or carboxylic acids with nucleophiles or radical precursors.^44–52^ However, these approaches often suffer from low product yield and require stoichiometric oxidants and harsh reaction conditions, limiting their applicability. Lactonization *via* photoredox catalysis remains rare.^53–55^ Surprisingly, during our investigations of the photoreductive coupling reactions, we discovered that lactones could be formed instead of tertiary alcohols when benzophenone was coupled with methyl acrylate. Although the yield of product P11 is only 47%, this unexpected result suggests a simple and mild alternative approach for synthesizing lactones, which may offer advantages over traditional methods. The influence of different substituent groups on the benzophenone substrate was carefully investigated and summarized in Scheme 4. The results exhibit a similar trend to the umpolung C–C coupling of ketones (Scheme 1), with electron-poor substrates outperforming those with electron-donating substituents. Notably, when the substrate S3, bearing an electron-withdrawing cyano group was used, a moderate yield of 51% was achieved. However, a significant drop in yield to 11% was observed when an electron-donating methyl group was introduced (S11), and no conversion could be observed with the even more electron-rich *para*-methoxy-substituted (S2) or 2-thienyl (S5) variants was used. Surprisingly, when phenyl(pyridin-2-yl)methanone (S4) was subjected to the reaction, the product of lactonization was not observed, instead the linear product of umpolung C–C coupling reaction was formed in 23% yield (P14). A proposed mechanistic pathway is given in Fig. S7,† and it seems that the 2-pyridyl group either quenches radical intermediate B that forms *via* C–C radical coupling or inhibits the cyclization rate. Nevertheless, the observation of the linear C–C bond formation product P14 under the same conditions that γ-lactones are commonly formed suggests that C–C bond formation precedes cyclization, as proposed in Fig. S7.†

### Mechanistic considerations

**Scheme 5:**
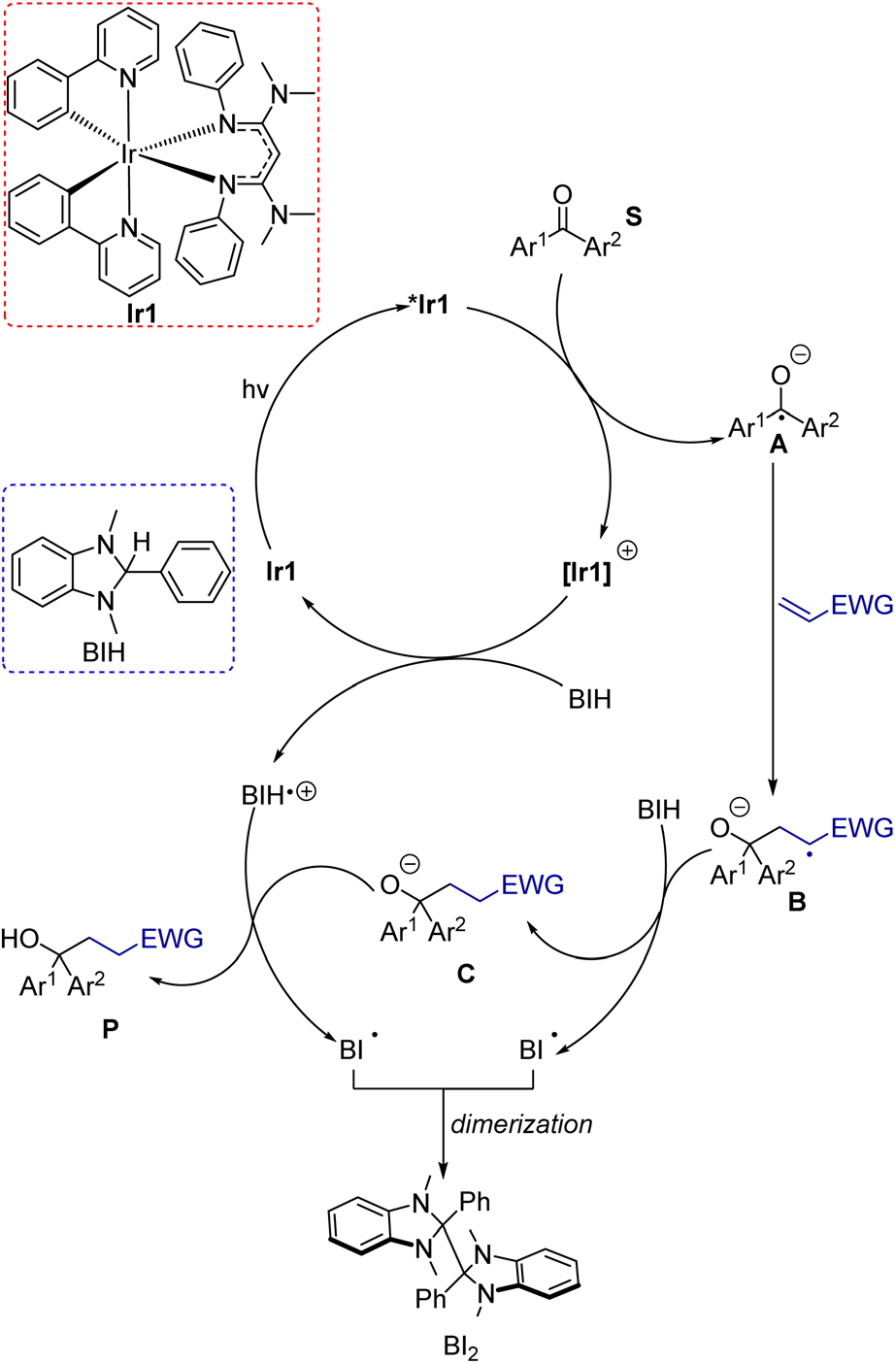
Proposed mechanism for umpolung C–C coupling.

To clarify mechanistic aspects of these transformations, control experiments were conducted, which are summarized in Table 1 (entries 12–15). In the absence of Ir1 (entry 12), in the dark (entry 13), or in air (entry 14), no coupling product was observed. These experiments clearly confirmed the important role of the photosensitizer and light in this transformation. Additionally, when we employed the radical scavenger 2,2,6,6-tetramethyl-1-piperidinyloxy (TEMPO), the reaction was completely suppressed, and no product was detected (entry 15). Although the trapping product of TEMPO was not isolated, this experiment suggests that these reactions likely proceed through a radical pathway. Previously reported Stern–Volmer quenching experiments of Ir1 with benzophenone (S1) showed that the excited-state electron transfer rate constant is 5.6(6) × 10^9^ M^−1^ s^−1^, a factor of 3 higher than that of *fac*-Ir(ppy)_3_ (Ir2, 1.9(2) × 10^9^ M^−1^ s^−1^).^14^ This experiment reveals that the excited state of Ir1 is capable of reducing benzophenone to generate the ketyl intermediate. The quenching rate of BIH with Ir1 was also previously measured and found to be much slower at 2.3 × 10^8^ M^−1^ s^−1^. Under reaction conditions with 2 equivalents of BIH present, quenching by benzophenone is still kinetically favored by a factor of 12. Based on these experiments and the relevant redox potentials, we propose that the primary photoreaction involves an oxidative quenching pathway. Taking these insights all together, a proposed mechanism for these reactions is shown in Scheme 5. Initially, Ir1 is excited by visible light to its ^3^MLCT excited state, abbreviated as *Ir1, which transfers an electron to the benzophenone substrate. Importantly, this electron-transfer step can occur efficiently without prior activation of the ketone or inclusion of additives. To close the photocycle, the oxidized [Ir1]^+^ is reduced back to Ir1 by BIH, which generates the BIH radical cation (BIH˙^+^). On the basis of their respective redox potentials, −0.26 V for Ir1 (ref. 40) and −0.07 V for BIH^56^ (both *vs.* ferrocene), we expect this step to be slightly endergonic. Ketyl intermediate A is formed in the primary photoreaction, which then participates in intermolecular coupling with the electron-poor alkene to produce intermediate B. Hydrogen atom transfer to B from BIH produces anion C, which then undergoes proton transfer with the BIH radical cation to from the product. Note that the sequential H-atom and proton transfer steps that convert B to the final product could occur in either order, and it is also possible that ketyl intermediate A is first protonated by BIH or BIH˙^+^ to form the neutral radical species prior to C–C bond formation. Although we have less direct insight into the dark reactions (bottom cycle in Scheme 5), BIH is known to be both a good reductant and a strong hydrogen atom donor,^56,57^ lending credence to its proposed roles in Scheme 5. In net two equivalents of BIH are involved, one of which undergoes H-atom transfer and the other sequential electron and proton transfer, both generating the radical BI˙. We did not attempt to isolate the product(s) derived from BIH, but it has been shown previously that dimerization of BI˙ can occur.^58^

In addition to the proposed mechanism mentioned above, there are other potential pathways that cannot be explicitly ruled out. One possibility involves reductive quenching with BIH as the primary photoreaction, although as mentioned above the pseudo-first order quenching rate of Ir1 with BIH is a factor of 12 lower than the quenching rate of Ir1 with benzophenone. This reductive quenching mechanism (Fig. S8†) could be a minor pathway, whereby the excited Ir1 is quenched by BIH and the substrate would be reduced by [Ir1]˙^−^ to produce the ketyl.

Given the high concentration of alkene in reaction, we also considered the possibility that the excited state of Ir1 could be quenched by the alkene coupling partner. Stern–Volmer quenching experiments of Ir1 with alkenes were conducted to measure rates of quenching by the alkenes (Fig. S9†). The experiments yielded quenching rate constants (*k*_q_) of 1.3 × 10^8^, 4.4 × 10^7^, and 6.3 × 10^7^ M^−1^ s^−1^ for phenyl vinyl sulfone, acrylonitrile, and methyl acrylate, respectively. Accounting for the 10-fold excess of alkene in the reactions, we still estimate that the quenching rate for the alkenes is a factor of 4.3–11× smaller than the quenching rate for benzophenone, again suggesting that the reductive quenching pathway shown in Scheme 5 is the kinetically preferred mechanism. That said, these rates are similar enough that a pathway involving a bimolecular reaction of the alkene with *Ir1 could be competitive, particularly at later time points. Provided there are no side reactions involving the alkene, as the ketone substrate depletes the alkene will be present in even larger excess relative to the ketone, which would make the quenching rates of the two substrates more similar as the reaction progresses.

One final mechanistic possibility involves triplet–triplet energy transfer between Ir1 and benzophenone. The triplet excited-state energy of benzophenone is well-characterized and has an energy of *ca.* 3.0 eV.^59^ The triplet energy of Ir1 is estimated to be 2.3 eV,^14^ so energy transfer would be significantly endergonic and thus unlikely. If it were to occur, the triplet benzophenone could be reduced by BIH to produce the ketyl and BIH˙+ intermediates. These intermediates would then follow a similar mechanism in Scheme 5 to generate the final product. Although various pathways are feasible for these reactions, thermodynamic analyses and Stern–Volmer quenching experiments point to the oxidative quenching mechanism illustrated in Scheme 5 as the most likely.

## Conclusion

In this work, we demonstrate versatile photoredox functionalization strategies of challenging carbonyl substrates, all involving C–C bond formation and operating under simple and uniform reaction conditions, without protecting groups or additives. These reactions are enabled by the efficient generation of ketyl and iminyl intermediates upon visible-light excitation of a strongly photoreducing iridium photosensitizer. Reactions classes demonstrated with this general approach include umpolung C–C bond forming reactions, imino-pinacol coupling, and γ-lactonization. Ketones and imines represent versatile building blocks for organic synthesis, but they have not been as extensively used in photoredox catalysis because they are challenging to reduce or oxidize. This work presents a significant advance in this regard and motivates further exploration of carbonyl functionalization reactions facilitated by strongly reducing photosensitizers.

## Experimental

### General procedure for photoredox catalysis

Additional details and all characterization data are supplied in the ESI.† In a typical photoredox reaction, a 10 mL DMF solution of Ir(ppy)_2_(NacNac^NMe2^) (Ir1, 1.8 mg) was prepared in a 20 mL vial. A 10 mL quantity of DMF was added to another 20 mL vial which had been charged with the ketone or imine substrate (0.2 mmol), BIH (2 equivalents), and, for C–C coupling reactions, the alkene (10 equivalents), and the mixture was stirred for 5 minutes. Then, the solution of Ir(ppy)_2_(NacNac^NMe2^) (Ir1) was added to the mixture, giving 1 mol% catalyst loading. The vial was sealed with a cap and parafilm and was taken out of the glovebox. The vial was irradiated with blue LED light (430–500 nm, *λ*_max_ = 463 nm) for 48 h. After the solvent was removed by rotary evaporation, 20 mL of ethyl acetate was added. A white solid precipitated which was removed by filtration. The filtrate was extracted with water and dried over MgSO_4_. Then, the solvent was removed under vacuum. The product was purified by silica gel column chromatography using ethyl acetate and hexane as the eluent. Finally, the product was dried under vacuum overnight and characterized by ^1^H NMR and ^13^C{^1^H} NMR.

## Data availability

The datasets supporting this article have been uploaded as part of the ESI.†

## Author contributions

Vinh Q. Dang: methodology, validation, formal analysis, investigation, writing – original draft, writing – review & editing, visualization. Thomas S. Teets: conceptualization, writing – review & editing, visualization, supervision, project administration, funding acquisition.

## Conflicts of interest

The authors declare no competing interests.

## Supplementary Material

SC-014-D3SC03000H-s001
