# Supplementary material for: Reductive photoredox transformations of carbonyl derivatives enabled by strongly reducing photosensitizers
Source: Chem Sci. 2023 Aug 18;14(35):9526–32. doi: 10.1039/d3sc03000h (PMC10498680; doi:10.1039/d3sc03000h)
Supplement: SC-014-D3SC03000H-s001 [file SC-014-D3SC03000H-s001.pdf]

Electronic Supplementary Material (ESI) for Chemical Science.
This journal is © The Royal Society of Chemistry 2023

Electronic Supplementary Information

Reductive Photoredox Transformations of Carbonyl Derivatives
Enabled by Strongly Reducing Photosensitizers

Vinh Q. Dang and Thomas S. Teets*

University of Houston, Department of Chemistry 3585 Cullen Blvd., Room 112,

Houston, TX USA 77204-5003. Email: tteets@uh.edu

Contents

Index	Page
Experimental setup for the photoredox catalysis	S2
Wavelength profile of blue LED light	S2
UV-vis absorption spectrum of **Ir1**	S3
Optimization of the alkene loading	S3
Cyclic voltammograms of **S9** and **S10**	S4
A proposed mechanism for lactonization reaction	S5
A proposed reductive quenching mechanism for umpolung C–C coupling	S6
Experimental Section: General procedure	S7
Experimental Section: Physical methods	S7
Experimental Section: Synthesis and characterization	S7–S14
Stern - Volmer quenching experiments	S14–S15
NMR spectra	S16–S32
References	S33

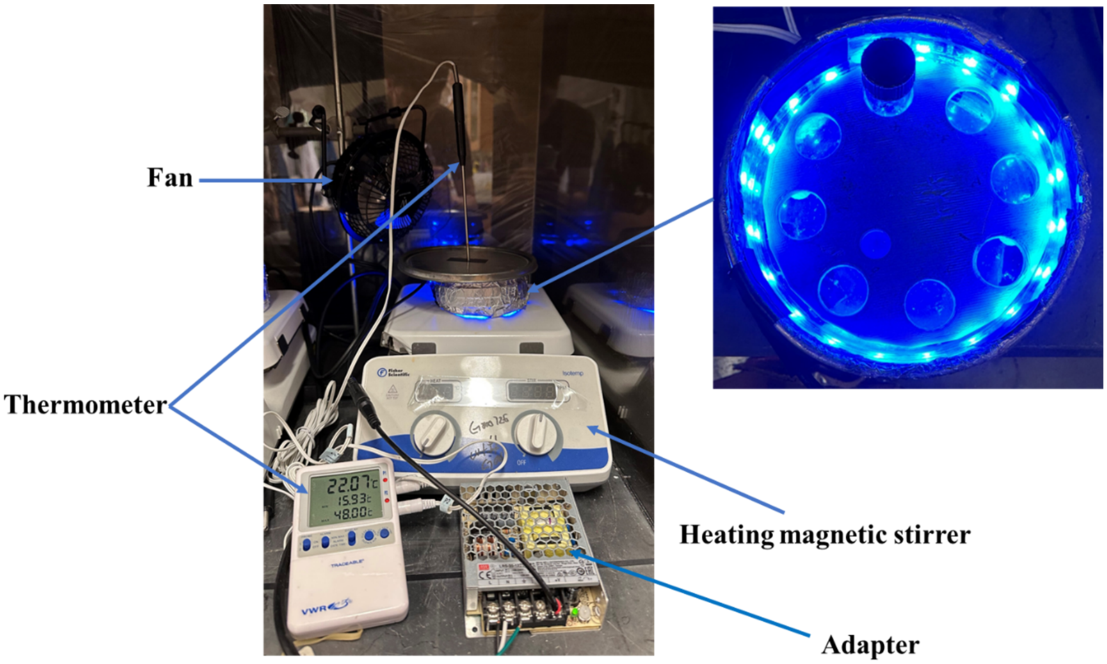

Fig. S1. Experimental setup for the photoredox catalysis.

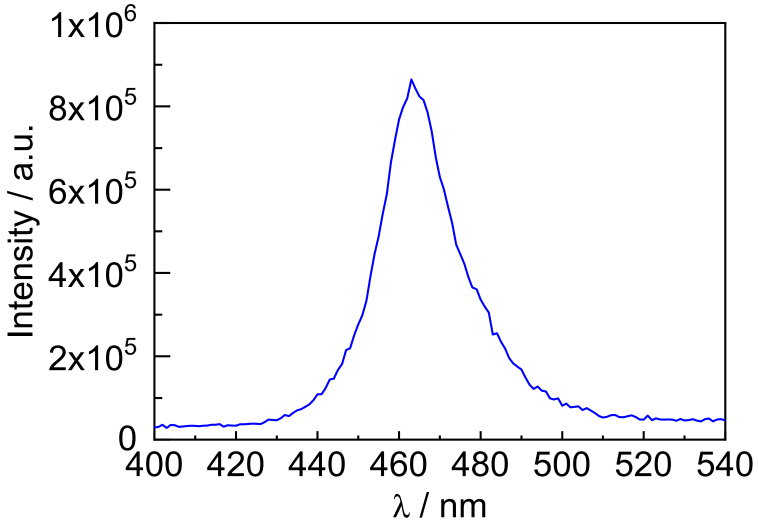

Fig. S2. Wavelength profile of the blue LED light used in photoredox experiments.

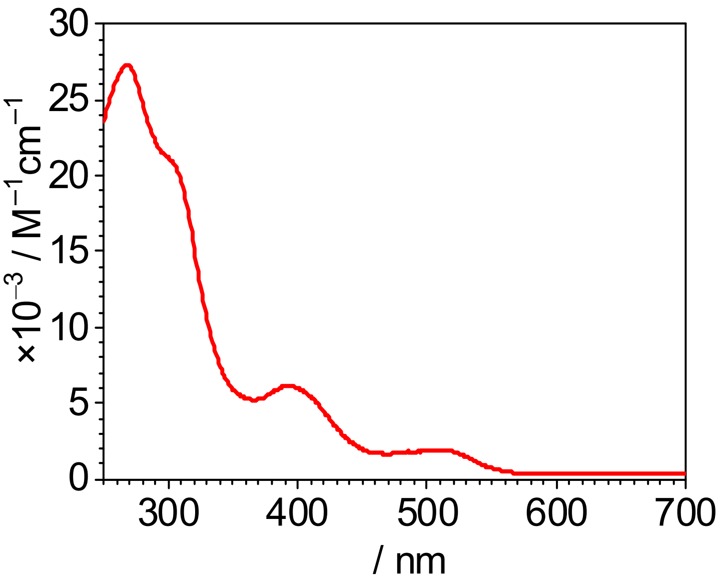

Fig. S3. UV-vis absorption spectrum of Ir(ppy)$_2$(NacNac^{NMe2}) (**Ir1**), recorded in THF. This spectrum was originally reported elsewhere.[1]

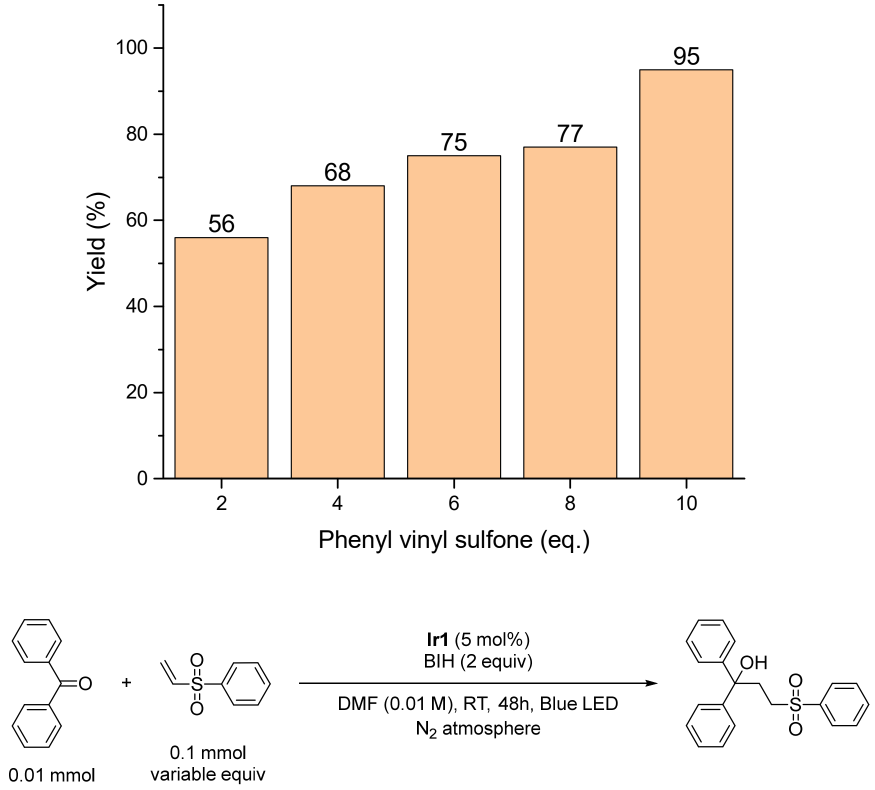

Fig. S4. Yield of umpolung C–C bond-formation as a function of phenyl vinyl sulfone equivalents.

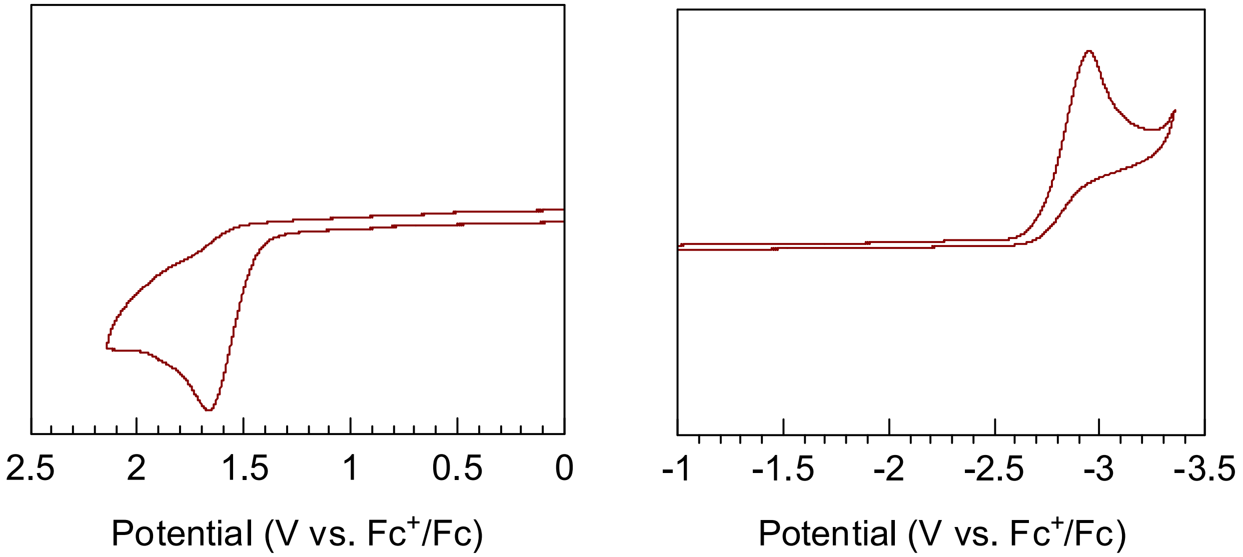

Fig. S5. Cyclic voltammograms of **S9**, recorded in MeCN with 0.1 M $(NBu_4)(PF_6)$ supporting electrolyte. Separate anodic (positive) and cathodic (negative) sweeps were recorded.

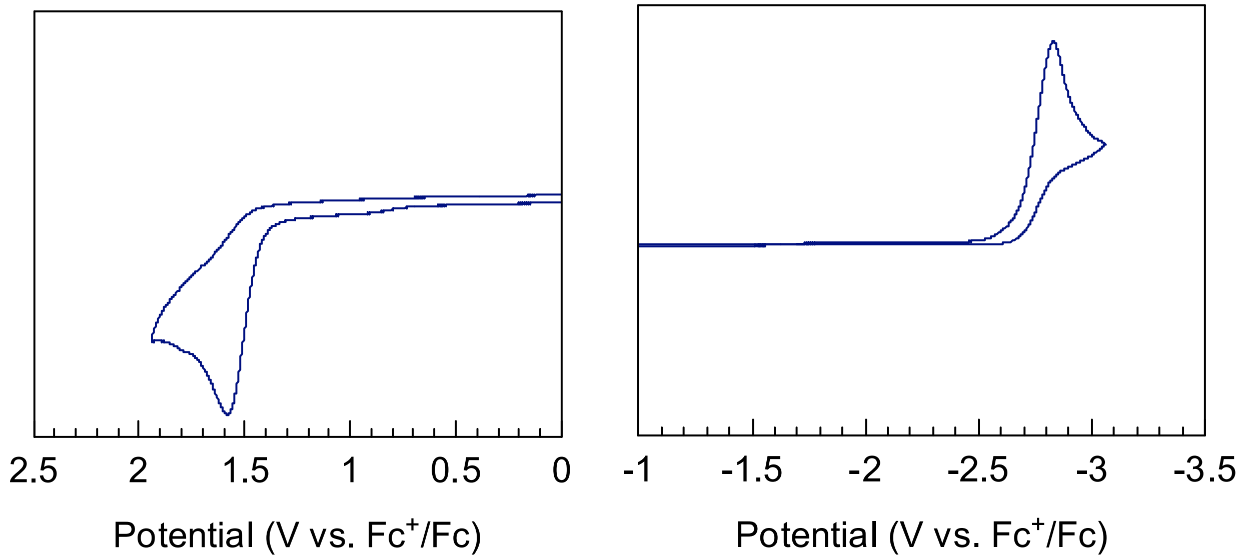

Fig. S6. Cyclic voltammograms of **S10**, recorded in MeCN with 0.1 M $(NBu_4)(PF_6)$ supporting electrolyte. Separate anodic (positive) and cathodic (negative) sweeps were recorded.

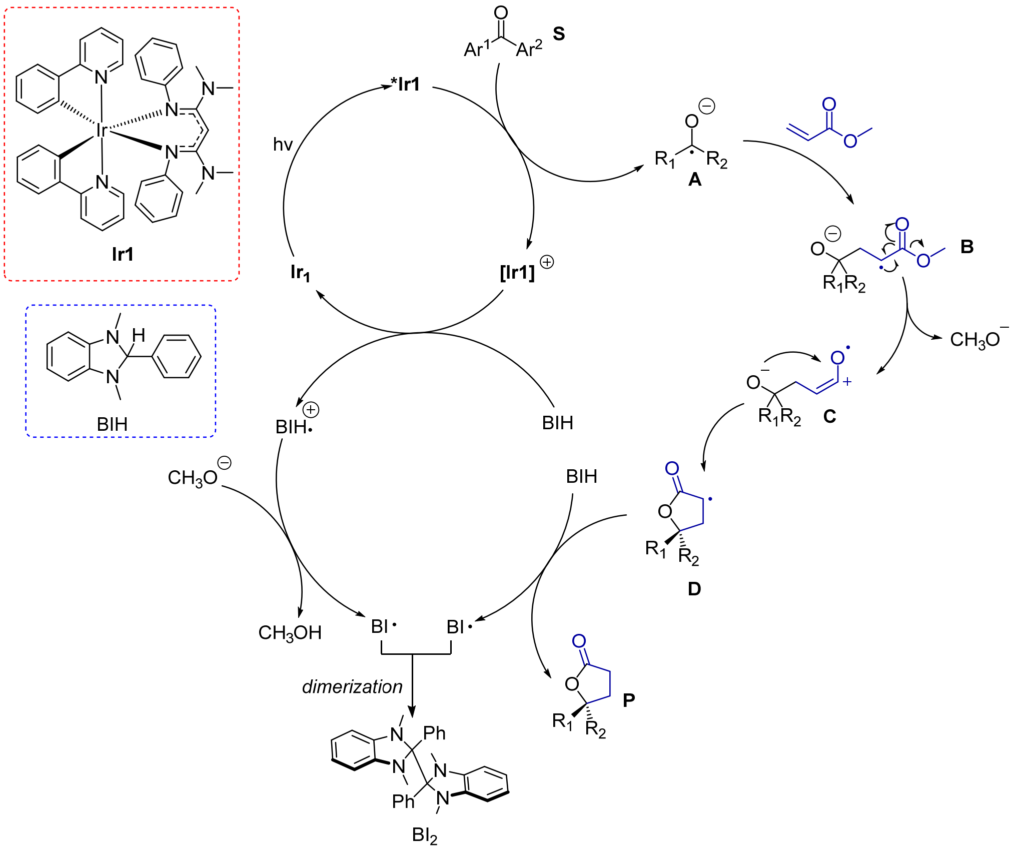

Fig. S7. A proposed mechanism for the lactonization reaction.

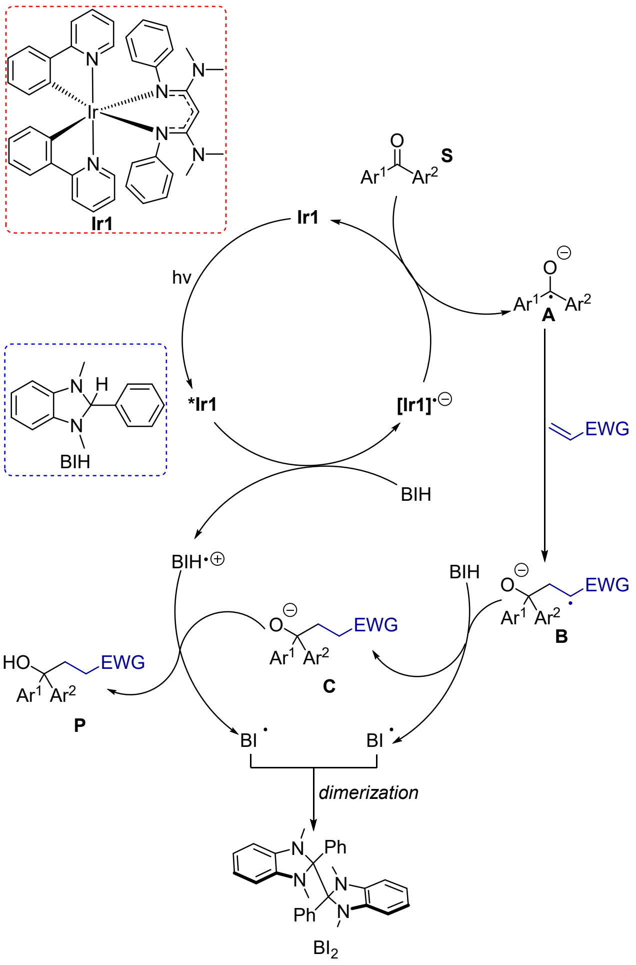

Fig. S8. A proposed reductive quenching mechanism for umpolung C–C coupling.

Experimental Section

Materials

Reagents for photoredox reactions were measured and combined in a nitrogen-filled glovebox. Anhydrous DMF was purchased from Sigma Aldrich and stored under an inert atmosphere. DMSO was degassed with the freeze-pump-thaw method and stored over 3 Å molecular sieves in a nitrogen-filled glovebox. All other solvents were dried by a commercial solvent purification system and stored over 3 Å molecular sieves. NMR solvents were purchased from Cambridge Isotope Laboratories. Starting materials and reagents were purchased from commercial suppliers (Sigma-Aldrich, TCI Chemicals, and AmBeed) and used without further purification unless otherwise specified. 1,3-dimethyl-2-phenyl-2,3-dihydro-1H-benzo[d]imidazole (BIH) was synthesized according to a reported literature[2]. $Ir(ppy)_2(NacNac^{NMe2})$ (**Ir1**)[3] and *fac*-$Ir(ppy)_3$ (**Ir2**)[4] were prepared according to previously described procedures. Tetrabutylammonium hexafluorophosphate, used as a supporting electrolyte for cyclic voltammetry experiments, was recrystallized from hot ethanol, and ferrocene, used as an internal standard for cyclic voltammetry experiments, was sublimed prior to use.

Physical Methods

^1H and $^{13}C\{^1H\}$NMR spectra (Fig. S8–S41) were recorded at room temperature using a JEOL ECA-400, JEOL ECA-500, or ECA-600 NMR spectrometer. Cyclic voltammograms were recorded using a CH Instruments 602E potentiostat interfaced with a nitrogen-filled glovebox. Samples were dissolved in MeCN with 0.1 M tetrabutylammonium hexafluorophosphate as the supporting electrolyte, and recorded using a glassy carbon working electrode, platinum wire counter electrode, and silver wire pseudoreference electrode. Ferrocene was added at the end of each measurement as an internal standard, and all potentials are referenced to the ferrocenium/ferrocene redox couple. Thin-layer chromatography was carried out using glass-backed silica gel plates. The visualization was achieved by irradiation under UV light (254nm or 366 nm). Column chromatography was performed on silica gel (230-400 mesh). Gas chromatography-mass spectrometry (GC-MS) was performed using an Agilent 7890 GC/5977A MSD instrument equipped with an HP-5MS capillary column. The temperature program for GC-MS analysis held samples at 50 °C for 5 min, then heated samples from 50 to 280 °C at 30 °C/min and held them at 280 °C for 18 min. Inlet temperature was set constant at 280 °C. Mass spectrograms were compared with the data gathered from the NIST library.

Synthesis and characterization

Preparation of the starting materials

N-**Benzylideneaniline (S8)**. 1.06 g (10.0 mmol) of benzaldehyde and 1.86 g (20.0 mmol) of aniline were dissolved to 10 mL of CH_2Cl_2 in a 20 mL scintillation vial, followed by addition of 5 g of $MgSO_4$. The mixture was stirred at room temperature for 4 h. Then, 10 mL of CH_2Cl_2 was added and solution was filtered. The resulting solution was extracted with water. The organic phase was collected and dried over $MgSO_4$. Then, solvent was removed by rotary evaporation. A yellow solid was obtained and washed with hexane. The final product was dried under vacuum overnight. (1.8

g, 98% yield). **^{1}H NMR** in $CDCl_3$ (400 MHz). δ 8.46 (s, 1H), 7.92–7.90 (m, 2H), 7.50–7.46 (m, 3H), 7.42–7.38 (m, 2H), 7.26–7.20 (m, 3H) ppm. **^{13}C{^{1}H} NMR** in $CDCl_3$ (101 MHz) δ 160.6, 152.2, 136.3, 131.5, 129.3, 128.9, 128.9, 126.1, 121.0 ppm.

***N*-*tert*-butyl-1-phenylmethanimine (S9)**. 1.06 g (10.0 mmol) of benzaldehyde and 1.4 g of *tert*-butylamine (20 mmol) were dissolved to 10 mL of DCM in a 50 mL rounded bottom flask, following by adding 5g of $MgSO_4$. The mixture was stirred at room temperature for 4h. Then, 10ml of DCM was added and the solution was filtered. The resulting solution was washed with water. The organic phase was collected and dried over $MgSO_4$. Then, solvent was removed by rotary evaporation. A colorless oil-like product was obtained and dried under vacuum overnight. (1.6 g, 98% yield). **^{1}H NMR** in $CDCl_3$ (400 MHz). δ 7.73 – 7.70 (m, 2H), 7.48 – 7.44 (m, 1H), 7.43 – 7.38 (m, 2H), 5.92 (s, 1H), 1.46 (s, 9H) ppm. **^{13}C{^{1}H} NMR** in $CDCl_3$ (101 MHz) δ 155.3, 137.2, 130.3, 128.6, 128.0, 57.3, 29.8 ppm.

Phenyl-*N*-propylmethanimine (S10). 1.06 g (10.0 mmol) of benzaldehyde and 1.2 g of *n*-propylamine (20.0 mmol) were dissolved to 10 mL of CH_2Cl_2 in a 50 mL round bottomed flask, following by addition of 5 g of $MgSO_4$. The mixture was stirred at room temperature for 4 h. Then, 10 mL of CH_2Cl_2 was added and the solution was filtered. The resulting solution was extracted with water. The organic phase was collected and dried over $MgSO_4$. Then, solvent was removed by rotary evaporation. A colorless oil-like product was obtained and dried under vacuum overnight. (1.4 g, 96% yield). **^{1}H NMR** in $CDCl_3$ (400 MHz). δ 8.27 (t, J = 1.3 Hz, 1H), 7.73–7.71 (m, 2H), 7.42–7.38 (m, 3H), 3.57 (td, *J* = 7.0, 1.4 Hz, 2H), 1.77–1.68 (m, 2H), 0.95 (t, *J* = 7.4 Hz, 3H) ppm. **^{13}C{^{1}H} NMR** in $CDCl_3$ (101 MHz) δ 161.0, 136.4, 130.6, 128.7, 128.1, 63.7, 24.2, 12.0 ppm.

General reaction procedures for photoredox catalysis

Procedure 1. Reaction screening. 20 mL DMF stock solutions of $Ir(ppy)_2(NacNac^{NMe2})$ (**Ir1**) and *fac*-$Ir(ppy)_3$ (**Ir2**) were prepared, at the appropriate concentrations to deliver 5 mol% or 1 mol% catalyst loading using 1 mL aliquots. Benzophenone (0.01 mmol), phenyl vinyl sulfone (variable equivalents), and sacrificial reagent (2 equiv relative to benzophenone) were added to 8 mL vials. Then, 1 mL of photocatalyst stock solution was distributed to each vial. The vial was sealed with a cap and parafilm and was taken out of the glovebox. The vial was irradiated with blue LED light (430–500 nm, λ_{max} = 463 nm) for 48 h. The reaction was tracked by using GC-MS with 2,4,6-trimethoxybenzene was used as the internal standard. The product peak in GC-MS was identified by comparison with isolated product, obtained after purification.

Procedure 2: Scale up procedure for determining the isolated yield. A 10 mL DMF solution of $Ir(ppy)_2(NacNac^{NMe2})$ (**Ir1**, 1.8 mg) was prepared in a 20 mL vial. A 10 mL quantity of DMF was added to another 20 mL vial which had been charged with the ketone or imine substrate, BIH (2 equivalents), and alkenes (10 equivalents), and the mixture was stirred for 5 minutes. Then, the solution of $Ir(ppy)_2(NacNac^{NMe2})$ (**Ir1**) was added to the mixture, giving 1 mol% catalyst loading. The vial was sealed with a cap and parafilm and was taken out of the glovebox. The vial was irradiated with blue LED light (430–500 nm, λ_{max} = 463 nm) for 48 h. After the solvent was removed by using rotary evaporation, 20 mL of ethyl acetate was added. A white solid precipitated

which was removed by filtration. The filtrate was extracted with water and dried over $MgSO_4$. Then, the solvent was removed under vacuum. The product was purified by silica gel column chromatography using ethyl acetate and hexane as an eluent. Finally, the product was dried under vacuum overnight and characterized by ^{1}H NMR and ^{13}C{^{1}H} NMR.

Detailed procedures for photoredox catalysis

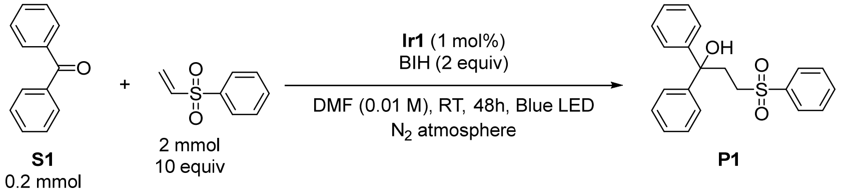

Synthesis of 1,1-diphenyl-3-(phenylsulfonyl)propan-1-ol (**P1**). The reaction was performed according to Procedure 2 described above, using benzophenone (36.4 mg, 0.200 mmol), 1 mol% **Ir1**, BIH (89.6 mg, 0.400 mmol), and phenyl vinyl sulfone (336 mg, 2.00 mmol). The product was isolated by silica gel column chromatography using ethyl acetate:*n*-hexane (9:1) as an eluent. Yield: 89%, white solid. **^{1}H NMR** in CD_2Cl_2 (400 MHz): δ 7.82–7.80 (m, 2H), 7.64 (tt, *J* = 7.4, 1.1 Hz, 1H), 7.56–7.51 (m, 2H), 7.32–7.25 (m, 8H), 7.23–7.19 (m, 2H), 3.09–3.05 (m, 2H), 2.67–2.63 (m, 2H), 2.39 (s, 1H) ppm. **^{13}C{^{1}H} NMR** in CD_2Cl_2 (101 MHz): δ 145.6, 139.2, 133.7, 129.4, 128.5, 127.9, 127.4, 125.7, 76.9, 51.9, 34.5 ppm.

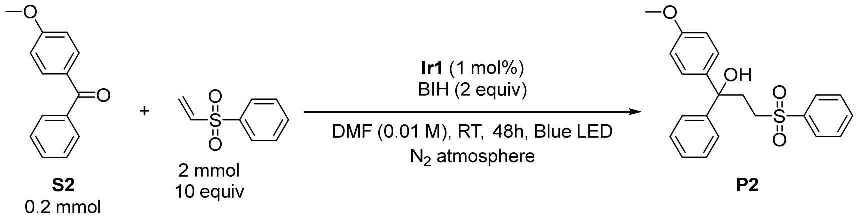

Synthesis of 1-(4-methoxyphenyl)-1-phenyl-3-(phenylsulfonyl)propan-1-ol (**P2**). The reaction was performed according to Procedure 2 described above, using (4-methoxyphenyl)(phenyl)methanone (42.4 mg, 0.200 mmol), 1 mol% **Ir1**, BIH (89.6 mg, 0.400 mmol), and phenyl vinyl sulfone (336 mg, 2.00 mmol). The product was isolated by silica gel column chromatography using ethyl acetate:*n*-hexane (8:2) as an eluent. Yield: 70%, white solid. **^{1}H NMR** in $CDCl_3$ (400 MHz): δ 7.86–7.83 (m, 2H), 7.63 (tt, *J* = 7.5, 1.3 Hz, 1H), 7.53 (t, *J* = 7.7 Hz, 2H), 7.29–7.28 (m, 4H), 7.24–7.19 (m, 3H), 6.82–6.78 (m, 2H), 3.77 (s, 3H), 3.17–3.01 (m, 2H), 2.73–2.62 (m, 2H), 2.19 (s, 1H) ppm. **^{13}C{^{1}H} NMR** in $CDCl_3$ (101 MHz): δ 158.9, 145.6, 139.2, 137.7, 133.8, 129.4, 128.6, 128.0, 127.5, 127.2, 125.8, 113.9, 55.4, 52.1, 34.5 ppm.

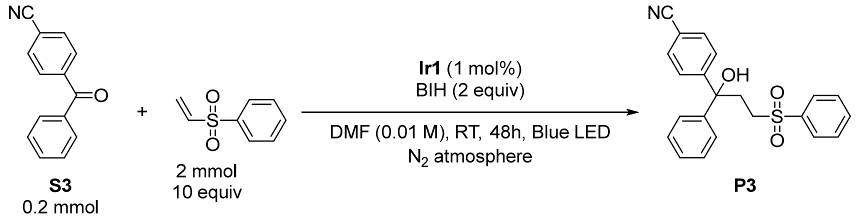

Synthesis of 4-(1-hydroxy-1-phenyl-3-(phenylsulfonyl)propyl)benzonitrile (**P3**). The reaction was performed according to Procedure 2 described above, using 4-benzoylbenzonitrile (41.4 mg, 0.200 mmol), 1 mol% **Ir1**, BIH (89.6 mg, 0.400 mmol), and phenyl vinyl sulfone (336 mg, 2.00 mmol). The product was isolated by silica gel column chromatography using ethyl acetate:*n*-hexane (7:3) as an eluent. Yield: 84%, white solid. **^{1}H NMR** in CD_3CN (400 MHz): δ 7.84–7.81 (m, 2H), 7.70 (tt, *J* = 7.5, 1.3 Hz, 1H), 7.61–7.56 (m, 4H), 7.48–7.45 (m, 2H), 7.32–7.24 (m, 4H), 7.22–7.18 (m, 1H), 4.01 (s, 1H), 3.10–2.93 (m, 2H), 2.65–2.53 (m, 2H) ppm. **^{13}C{^{1}H} NMR** in CD_3CN (101 MHz): δ 151.6, 145.3, 139.0, 134.0, 132.3, 129.5, 128.5, 128.0, 127.5, 126.6, 125.7, 118.7, 110.6, 76.2, 51.4, 34.0 ppm.

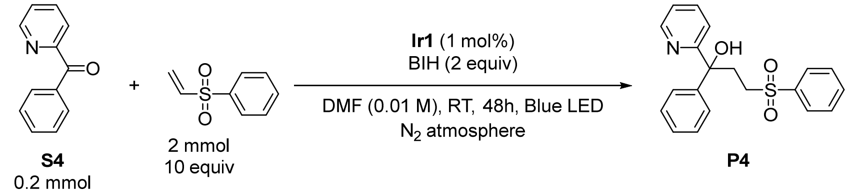

Synthesis of 1-phenyl-3-(phenylsulfonyl)-1-(pyridin-2-yl)propan-1-ol (**P4**). The reaction was performed according to Procedure 2 described above, using phenyl(pyridin-2-yl)methanone (36.6 mg, 0.200 mmol), 1 mol% **Ir1**, BIH (89.6 mg, 0.400 mmol), and phenyl vinyl sulfone (336 mg, 2.00 mmol). The product was isolated by silica gel column chromatography using ethyl acetate:*n*-hexane (9:1) as the eluent. Yield: 81%, yellow liquid. **^{1}H NMR** in $CDCl_3$ (500 MHz): δ 8.46 (d, *J* = 4.1 Hz, 1H), 7.87–7.85 (m, 2H), 7.65–7.59 (m, 2H), 7.52 (t, *J* = 7.9 Hz , 2H), 7.45 (d, *J* = 7.2 Hz, 2H), 7.29 (t, *J* = 7.7 Hz , 3H), 7.25–7.16 (m, 2H), 6.06 (s, 1H), 3.29–3.21 (m, 1H), 3.09–3.03 (m, 1H), 2.77–2.71 (m, 1H), 2.61–2.55 (m, 1H) ppm. **^{13}C{^{1}H} NMR** in $CDCl_3$ (126 MHz): δ 161.9, 147.6, 144.5, 139.2, 137.7, 133.8, 129.4, 128.7, 128.1, 127.6, 125.8, 122.8, 120.4, 76.1, 52.1, 33.9 ppm.

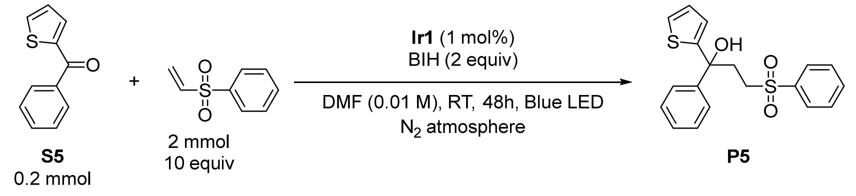

Synthesis of 1-phenyl-3-(phenylsulfonyl)-1-(thiophen-2-yl)propan-1-ol (**P5**). The reaction was performed according to Procedure 2 described above, using phenyl(thiophen-2-yl)methanone (37.6 mg, 0.200 mmol), 1 mol% **Ir1**, BIH (89.6 mg, 0.400 mmol), and phenyl vinyl sulfone (336 mg, 2.00 mmol). The product was isolated by silica gel column chromatography using ethyl acetate:*n*-hexane (8:2) as the eluent. Yield: 70%, white solid, **^{1}H NMR** in CD_3CN (500 MHz): δ 7.83–7.81 (m, 2H), 7.70 (tt, *J* = 7.4, 1.3 Hz, 1H), 7.58 (t, *J* = 7.8 Hz, 2H), 7.35–7.33 (m, 2H), 7.29–

7.25 (m, 2H), 7.23–7.20 (m, 2H), 6.89–6.87 (m, 2H), 4.17 (s, 1H), 3.21–3.15 (m, 1H), 2.94–2.88 (m, 1H), 2.61–2.52 (m, 2H) ppm. **^{13}C{^{1}H} NMR** in CD_3CN (126 MHz): δ 152.2, 145.2, 139.1, 134.0, 129.5, 128.3, 128.0, 127.4, 126.8, 125.2, 125.2, 123.7, 75.3, 51.6, 35.9 ppm.

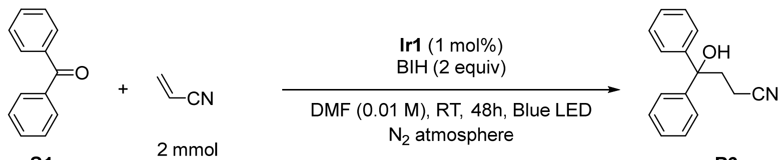

S1
0.2 mmol
P6

Synthesis of 4-hydroxy-4,4-diphenylbutanenitrile (**P6**). The reaction was performed according to Procedure 2 described above, using benzophenone (36.4 mg, 0.200 mmol), 1 mol% **Ir1**, BIH (89.6 mg, 0.400 mmol), and acrylonitrile (106 mg, 2.00 mmol). The product was isolated by silica gel column chromatography using ethyl acetate:*n*-hexane (8:2) as the eluent. Yield: 34%, colorless liquid. **^{1}H NMR** in $CDCl_3$ (400 MHz): δ 7.37–7.26 (m, 10H), 2.68–2.64 (m, 2H), 2.31–2.28 (m, 2H), 2.13 (br, s, 1H) ppm. **^{13}C{^{1}H} NMR** in $CDCl_3$ (101 MHz): δ 145.2, 128.7, 127.8, 126.0, 120.3, 77.3, 37.8, 12.3 ppm.

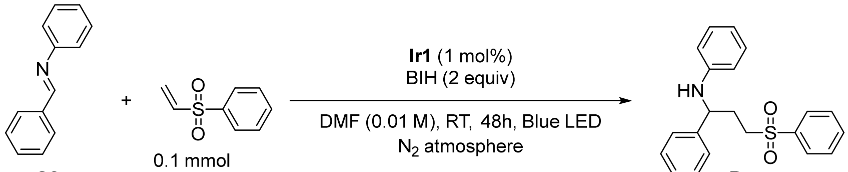

S8
0.2 mmol
P7

Synthesis of *N*-(1-phenyl-3-(phenylsulfonyl)propyl)aniline (**P7**). The reaction was performed according to Procedure 2 described above, using *N*-benzylideneaniline (36.2 mg, 0.200 mmol), 1 mol% **Ir1**, BIH (89.6 mg, 0.400 mmol), and phenyl vinyl sulfone (336 mg, 2.00 mmol). The product was isolated by silica gel column chromatography using ethyl acetate:*n*-hexane (8:2) as the eluent. Yield: 52%, brown solid. **^{1}H NMR** in CD_3CN (400 MHz): δ 7.85–7.83 (m, 2H), 7.69 (tt, *J* = 7.5, 1.3 Hz, 1H), 7.61–7.56 (m, 2H), 7.26–7.24 (m, 4H), 7.21–7.16 (m, 1H), 7.00–6.95 (m, 2H), 4.92 (d, *J* = 8.4 Hz, 1H), 4.42 (td, *J* = 8.4, 5.7 Hz 1H), 3.36–3.29 (m, 1H), 3.21–3.14 (m, 1H), 2.09–1.94 (m, 2H) ppm. **^{13}C{^{1}H} NMR** in CD_3CN (101 MHz): δ 147.6, 143.3, 139.2, 133.9, 129.5, 129.0, 128.7, 128.0, 127.3, 126.5, 117.1, 113.4, 55.8, 53.0, 31.1 ppm.

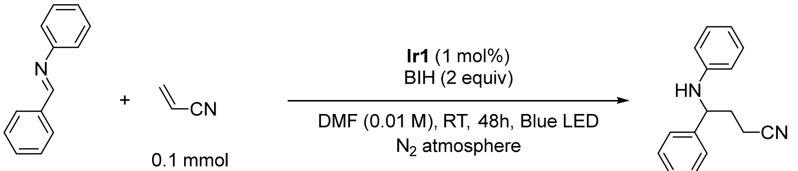

S8
0.2 mmol
P8

Synthesis of 4-phenyl-4-(phenylamino)butanenitrile (**P8**). The reaction was performed according to Procedure 2 described above, using *N*-benzylideneaniline (36.2 mg, 0.200 mmol), 1 mol% **Ir1**, BIH (89.6 mg, 0.400 mmol), and acrylonitrile (106 mg, 2.00 mmol). The product was

isolated by silica gel column chromatography using ethyl acetate:*n*-hexane (9:1) as the eluent. Yield: 43%, colorless liquid. ^{1}**H NMR** in $CDCl_3$ (400 MHz): δ 7.36–7.26 (m, 5H), 7.15–7.10 (m, 2H), 6.70 (tt, *J* = 7.3, 1.1 Hz, 1H), 6.58 (dt, *J* = 7.8, 1.0 Hz, 2H), 4.50 (t, *J* = 7.0 Hz, 1H), 4.04 (s, 1H), 2.49–2.31 (m, 2H), 2.23–2.06 (m, 2H). ^{13}**C{^{1}H} NMR** in $CDCl_3$ (101 MHz): δ 146.7, 141.7, 129.4, 129.2, 128.0, 126.4, 119.5, 118.2, 113.8, 57.0, 33.7, 14.6 ppm.

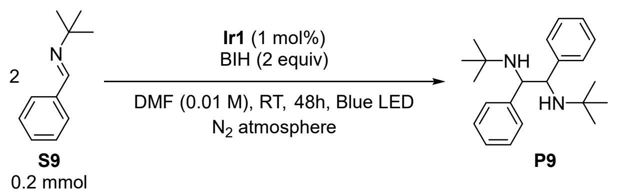

Synthesis of *N^{1}*,*N^{2}*-di-*tert*-butyl-1,2-diphenylethane-1,2-diamine (P9). The reaction was performed according to Procedure 2 described above, using *tert*-butyl-1-phenylmethanimine (32.2 mg, 0.200 mmol), 1 mol% **Ir1**, and BIH (89.6 mg, 0.400 mmol). The product was isolated by silica gel column chromatography using ethyl acetate: *n*-hexane (9:1) as an eluent. Yield: 27%, colorless liquid. ^{1}**H NMR** in CD_3CN (400 MHz): δ 7.18–7.15 (m, 4H), 7.10 7.06 (m, 4H), 7.05–7.01 (m, 2H), 3.66 (s, 2H), 1.24 (s, 2H), 0.80 (s, 18H) ppm. ^{13}**C{^{1}H} NMR** in $CDCl_3$ (101 MHz): δ 145.7, 127.8, 127.7, 126.2, 64.2, 51.0, 30.0 ppm.

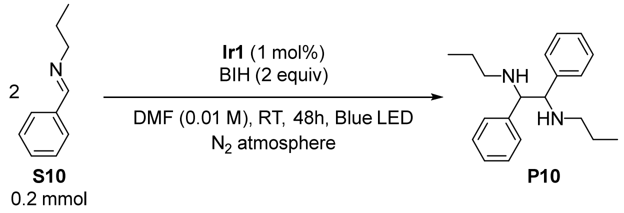

Synthesis of 1,2-diphenyl-*N^{1}*,*N^{2}*-dipropylethane-1,2-diamine (P10). The reaction was performed according to Procedure 2 described above, using 1-phenyl-*N*-propylmethanimine (29.4 mg, 0.2 mmol), 1 mol% $Ir(ppy)_2(NacNac^{NMe2})$, BIH (89.6 mg, 0.4 mmol). The product was isolated by silica gel column chromatography using ethyl acetate: *n*-hexane (9:1) as an eluent. Yield: 31%, colorless solid, ^{1}**H NMR** in $CDCl_3$ (400 MHz) δ 7.32 – 7.26 (m, 10H), 3.73 (s, 2H), 2.27 – 2.15 (m, 4H), 1.39 – 1.21 (m, 6H), 0.66 (t, J = 7.4 Hz, 6H) ppm. ^{13}**C NMR** in $CDCl_3$ (101 MHz) δ 141.4, 128.5, 128.4, 127.6, 68.5, 49.4, 22.8, 11.6 ppm.

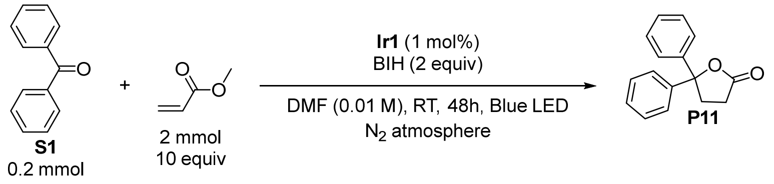

Synthesis of 5,5-diphenyldihydrofuran-2(3*H*)-one (**P11**). The reaction was performed according to Procedure 2 described above, using benzophenone (36.4 mg, 0.200 mmol), 1 mol% **Ir1**, BIH (89.6 mg, 0.400 mmol), and methyl acrylate (172 mg, 2.00 mmol). The product was isolated by silica gel column chromatography using ethyl acetate:*n*-hexane (9:1) as the eluent. Yield: 47%,

colorless liquid. **^{1}H NMR** in $CDCl_3$ (600 MHz): δ 7.42–7.40 (m, 4H), 7.35–7.32 (m, 4H), 7.28–7.25 (m, 2H), 2.90 (t, J = 7.8 Hz, 2H), 2.57 (t, J = 7.8 Hz, 2H) ppm. **$^{13}C\{^1H\}$ NMR** in $CDCl_3$ (101 MHz): δ 176.2, 143.1, 128.7, 128.0, 125.5, 89.8, 35.8, 29.1 ppm.

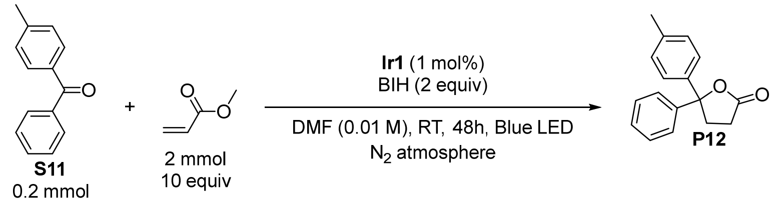

Synthesis of 5-phenyl-5-(*p*-tolyl)dihydrofuran-2(3*H*)-one (**P12**). The reaction was performed according to Procedure 2 described above, using phenyl(*p*-tolyl)methanone (39.2 mg, 0.200 mmol), 1 mol% **Ir1**, BIH (89.6 mg, 0.400 mmol), and methyl acrylate (172 mg, 2.00 mmol). The product was isolated by silica gel column chromatography using ethyl acetate:*n*-hexane (9:1) as the eluent. Yield: 11%, colorless liquid, **^{1}H NMR** in CD_3CN (500 MHz): δ 7.40–7.37 (m, 2H), 7.35–7.31 (m, 2H), 7.29–7.24 (m, 3H), 7.15 (d, J = 7.5 Hz, 2H), 2.94–2.84 (m, 2H), 2.47 (t, J = 7.7 Hz, 2H), 2.27 (s, 3H) ppm. **$^{13}C\{^1H\}$ NMR** in CD_3CN (126 MHz): δ 176.2, 144.0, 140.8, 137.8, 129.2, 128.6, 127.7, 125.3, 125.3, 89.4, 34.9, 28.7, 20.1 ppm.

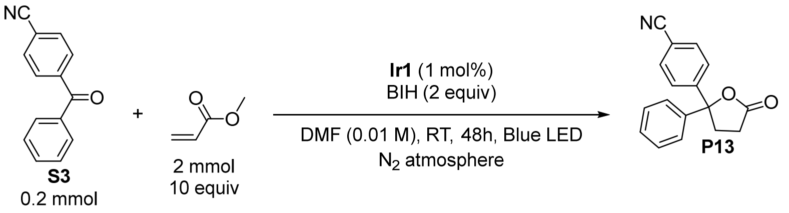

Synthesis of 4-(5-oxo-2-phenyltetrahydrofuran-2-yl)benzonitrile (**P13**). The reaction was performed according to Procedure 2 described above, using 4-benzoylbenzonitrile (41.4 mg, 0.200 mmol), 1 mol% **Ir1**, BIH (89.6 mg, 0.400 mmol), and methyl acrylate (172 mg, 2.00 mmol). The product was isolated by silica gel column chromatography using ethyl acetate:*n*-hexane (8:2) as the eluent. Yield: 51%, colorless liquid. **^{1}H NMR** in $CDCl_3$ (500 MHz). δ 7.65–7.63 (m, 2H), 7.55–7.53 (m, 2H), 7.41–7.35 (m, 4H), 7.33–7.29 (m, 1H), 3.03–2.98 (m, 1H), 2.85–2.79 (m, 1H), 2.65–2.54 (m, 2H) ppm. **$^{13}C\{^1H\}$ NMR** in $CDCl_3$ (126 MHz) δ 175.3, 148.4, 141.6, 132.6, 129.1, 128.6, 126.2, 125.4, 118.4, 112.1, 88.9, 35.5, 28.9 ppm.

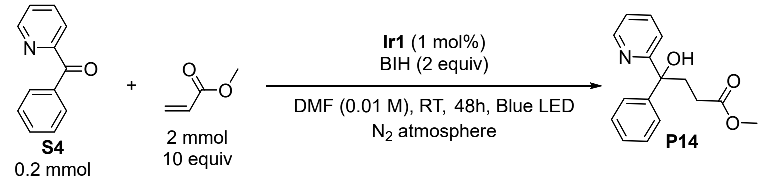

Synthesis of methyl 4-hydroxy-4-phenyl-4-(pyridin-2-yl)butanoate (**P14**).

The reaction was performed according to Procedure 2 described above, using phenyl(pyridin-2-yl)methanone (36.6 mg, 0.200 mmol), 1 mol% **Ir1**, BIH (89.6 mg, 0.400 mmol), and methyl acrylate (172 mg, 2.00 mmol). The product was isolated by silica gel column chromatography using ethyl acetate:*n*-hexane (8:2) as the eluent. Yield: 23%, white solid, **^{1}H NMR** in $CDCl_3$ (400

MHz): δ 8.49 (d, J = 5.0 Hz, 1H), 7.65 (td, J = 7.7, 1.8 Hz, 1H), 7.54–7.51 (m, 2H), 7.36–7.29 (m, 3H), 7.23–7.15 (m, 2H), 6.02 (s, 1H), 3.61 (s, 3H), 2.73–2.66 (m, 1H), 2.60–2.52 (m, 1H), 2.48–2.40 (m, 1H), 2.31–2.23 (m, 1H) ppm. **^{13}C{^{1}H} NMR** in $CDCl_3$ (126 MHz): δ 174.6, 163.0, 147.4, 145.6, 137.4, 128.5, 127.2, 126.0, 122.4, 120.6, 76.6, 51.7, 36.0, 29.1 ppm.

Stern-Volmer quenching experiments

Time-resolved quenching experiment:

Procedure: In the glovebox, stock solutions of **Ir1** (2 mg in 1 mL of DMF), phenyl vinyl sulfone (30 mg in 1 mL of DMF), acrylonitrile (20 mg in 1 mL of DMF), and methyl acrylate (20 mg in 1 mL of DMF) were prepared. The cuvette was filled with 3 mL of DMF and 10–15 µL of the stock solution of **Ir1**. The photoluminescence lifetime of the solution was recorded in the absence of quencher and after each addition of 5 µL aliquots of the quencher solution. The excitation wavelength was 453 nm.

Quenching rate calculation.

Ir1 with phenyl vinyl sulfone:

Concentration (M)	Lifetime (s)	τ_0/τ_q
0	7.10×10^{-7}	1.00
0.000299	6.89×10^{-7}	1.03
0.000597	6.67×10^{-7}	1.06
0.000896	6.52×10^{-7}	1.09
0.00119	6.38×10^{-7}	1.11
0.00149	6.24×10^{-7}	1.14

Ir1 with acrylonitrile:

Concentration (M)	Lifetime (s)	τ_0/τ_q
0	6.97×10^{-7}	1.00
0.00000377	6.72×10^{-7}	1.04
0.00000566	6.59×10^{-7}	1.06
0.00000755	6.49×10^{-7}	1.07
0.00000943	6.37×10^{-7}	1.09
0.00001132	6.24×10^{-7}	1.12

Ir1 with methyl acrylate:

Concentration (M)	Lifetime (s)	τ_0/τ_q
0	6.84×10^{-7}	1.00
0.00000116	6.68×10^{-7}	1.02
0.00000233	6.57×10^{-7}	1.04
0.00000349	6.50×10^{-7}	1.05
0.00000465	6.39×10^{-7}	1.07
0.00000581	6.28×10^{-7}	1.09
0.00000698	6.21×10^{-7}	1.10

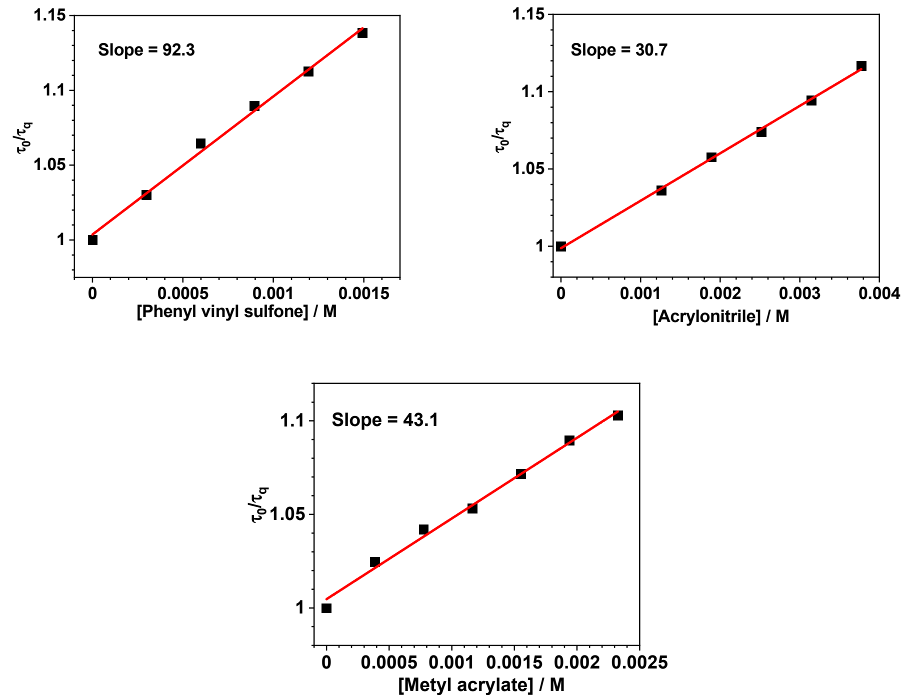

**Quencher**	**Slope** ($k_q \times \tau_0$)	$\boldsymbol{\tau_0}$ (s)	$\boldsymbol{k_q}$ (M^{-1} s^{-1})
Phenyl vinyl sulfone	92.3	7.10×10^{-7}	1.3×10^{8}
Acrylonitrile	30.7	6.97×10^{-7}	4.4×10^{7}
Methyl acrylate	43.1	6.84×10^{-7}	6.3×10^{7}

Fig. S9. Stern-Volmer quenching plots for **Ir1** with alkenes.

NMR Spectra.

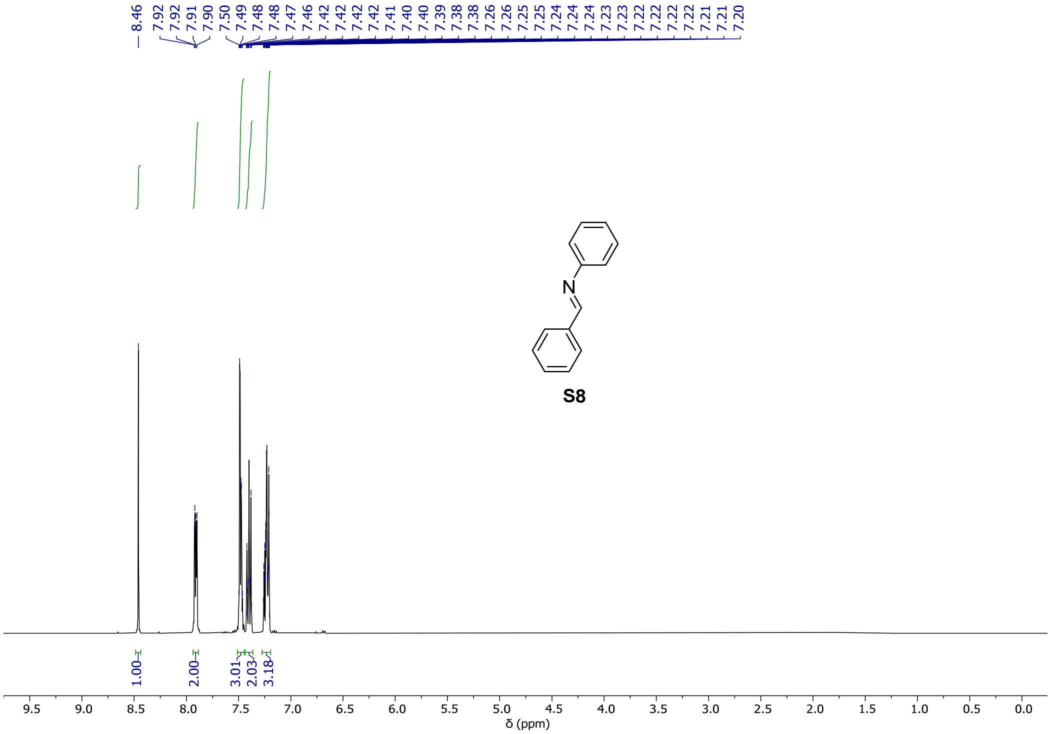

Fig. S10. ^{1}H NMR spectrum of **S8**, recorded in $CDCl_3$ at 400 MHz.

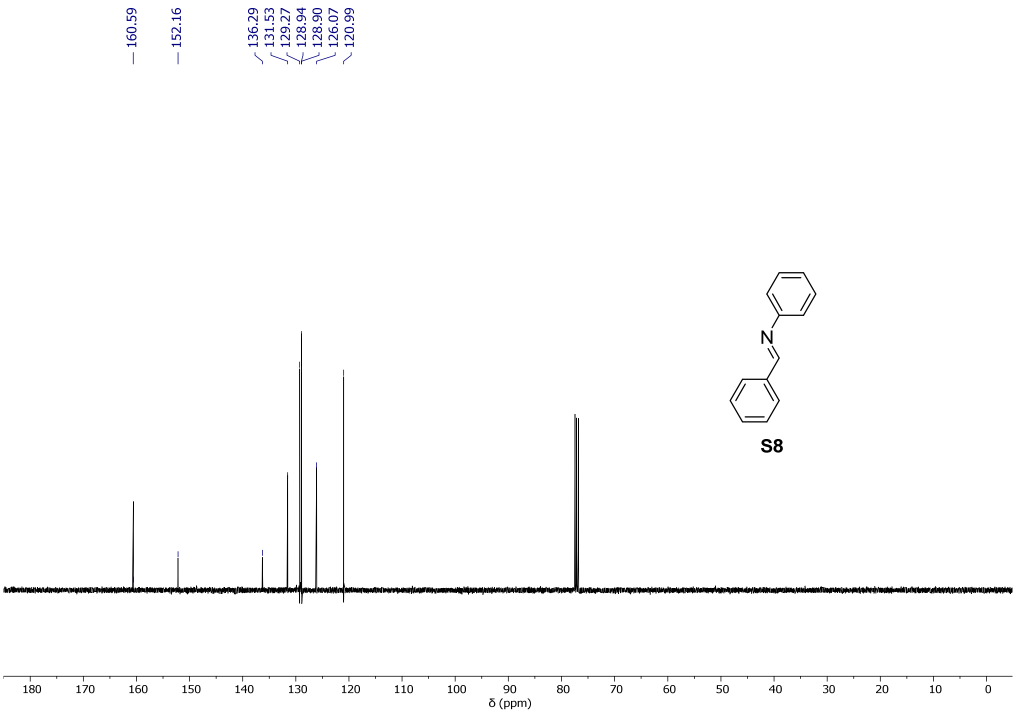

Fig. S11. $^{13}C\{^{1}H\}$ NMR spectrum of **S8**, recorded in $CDCl_3$ at 101 MHz.

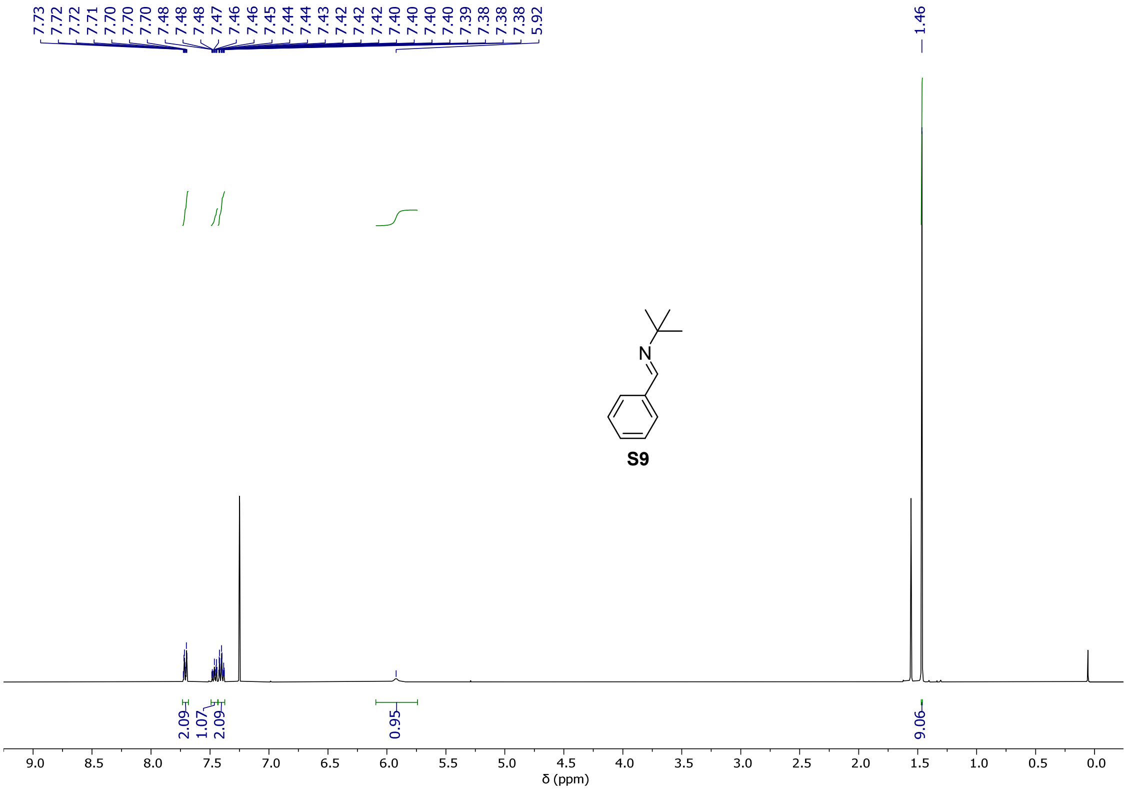

Fig. S12. ^{1}H NMR spectrum of **S9**, recorded in $CDCl_3$ at 400 MHz.

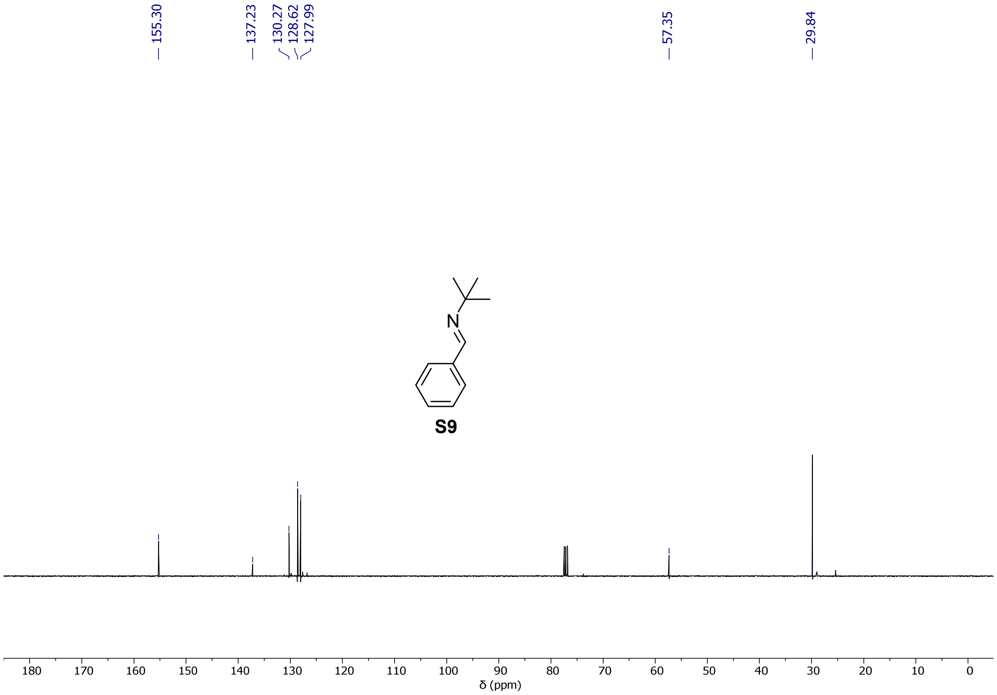

Fig. S13. $^{13}C\{^{1}H\}$ NMR spectrum of **S9**, recorded in $CDCl_3$ at 101 MHz.

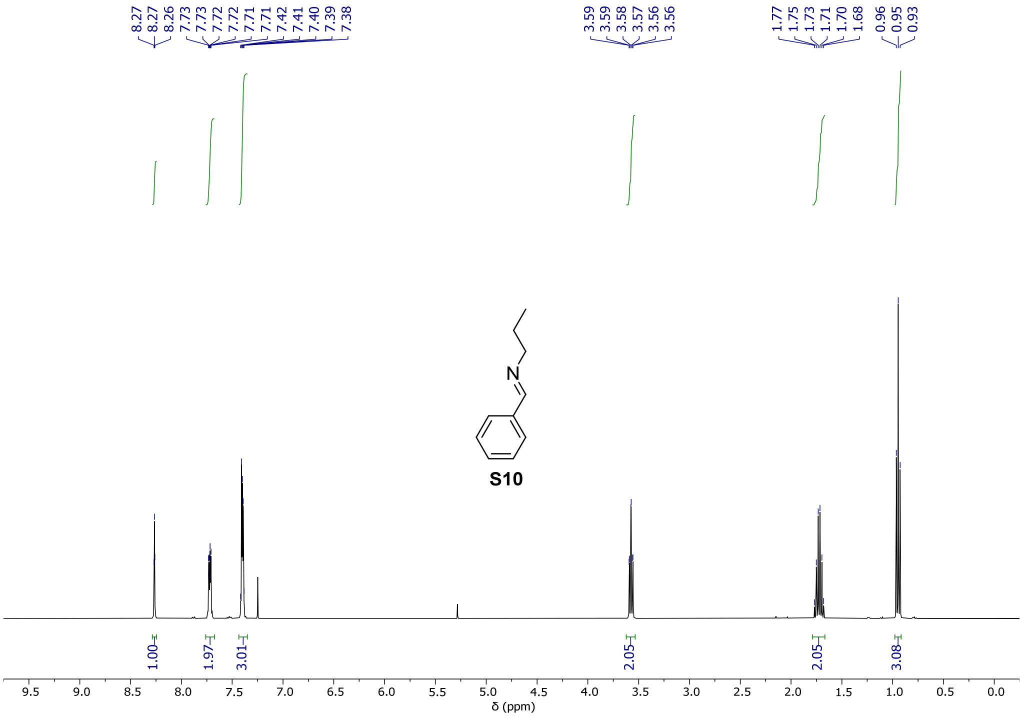

Fig. S14. ^{1}H NMR spectrum of **S10**, recorded in $CDCl_3$ at 400 MHz.

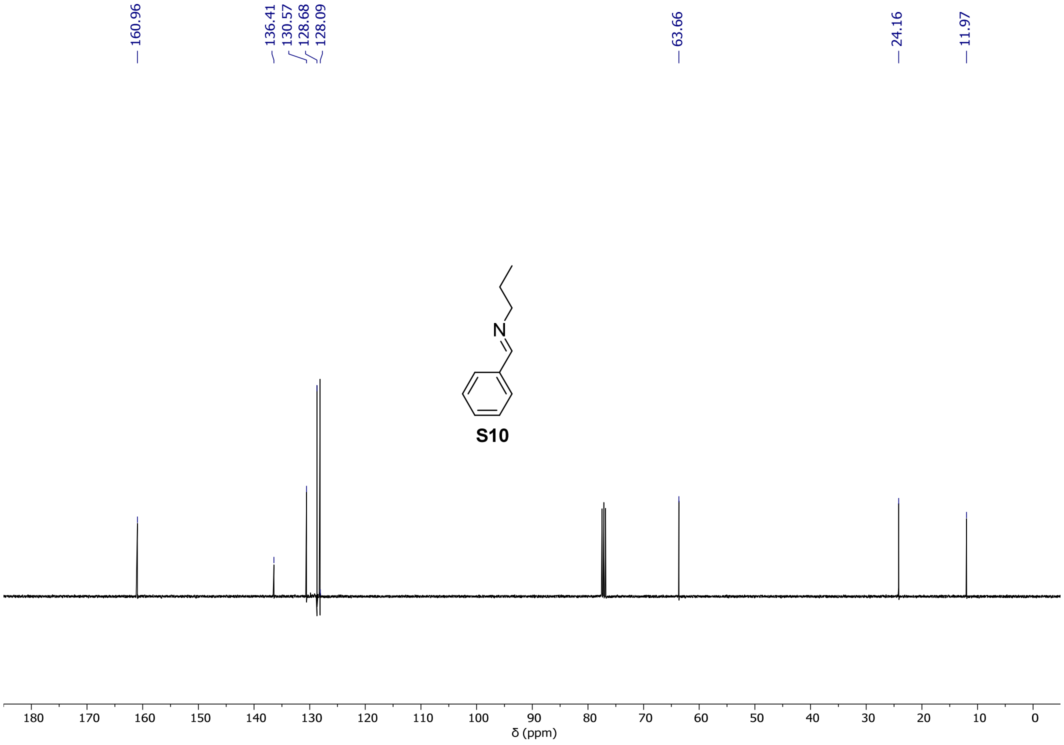

Fig. S15. $^{13}C\{^{1}H\}$ NMR spectrum of **S10**, recorded in $CDCl_3$ at 101 MHz.

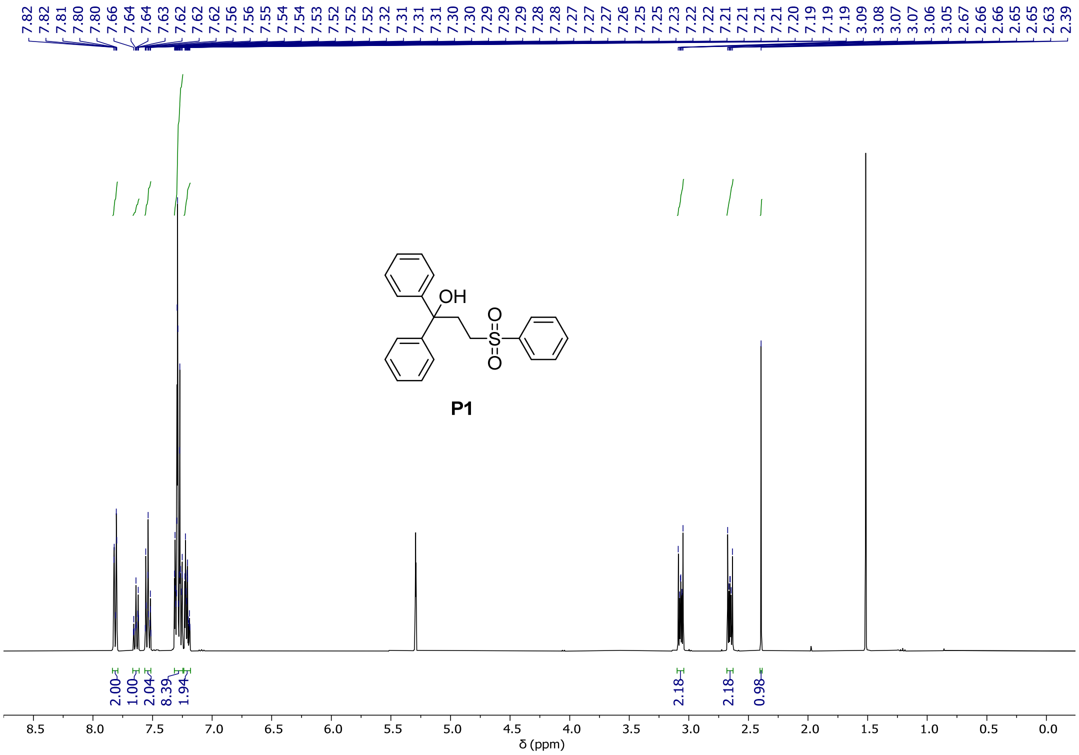

Fig. S16. ^{1}H NMR spectrum of **P1**, recorded in CD_2Cl_2 at 400 MHz.

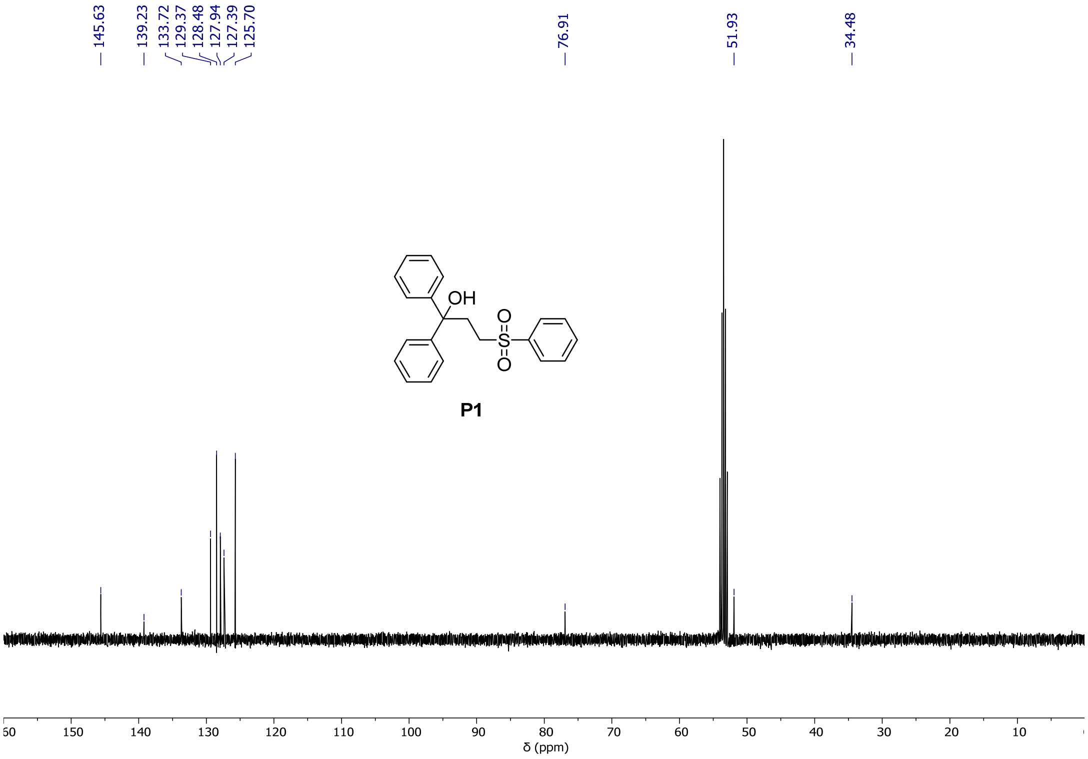

Fig. S17. $^{13}C\{^{1}H\}$ NMR spectrum of **P1**, recorded in CD_2Cl_2 at 101 MHz.

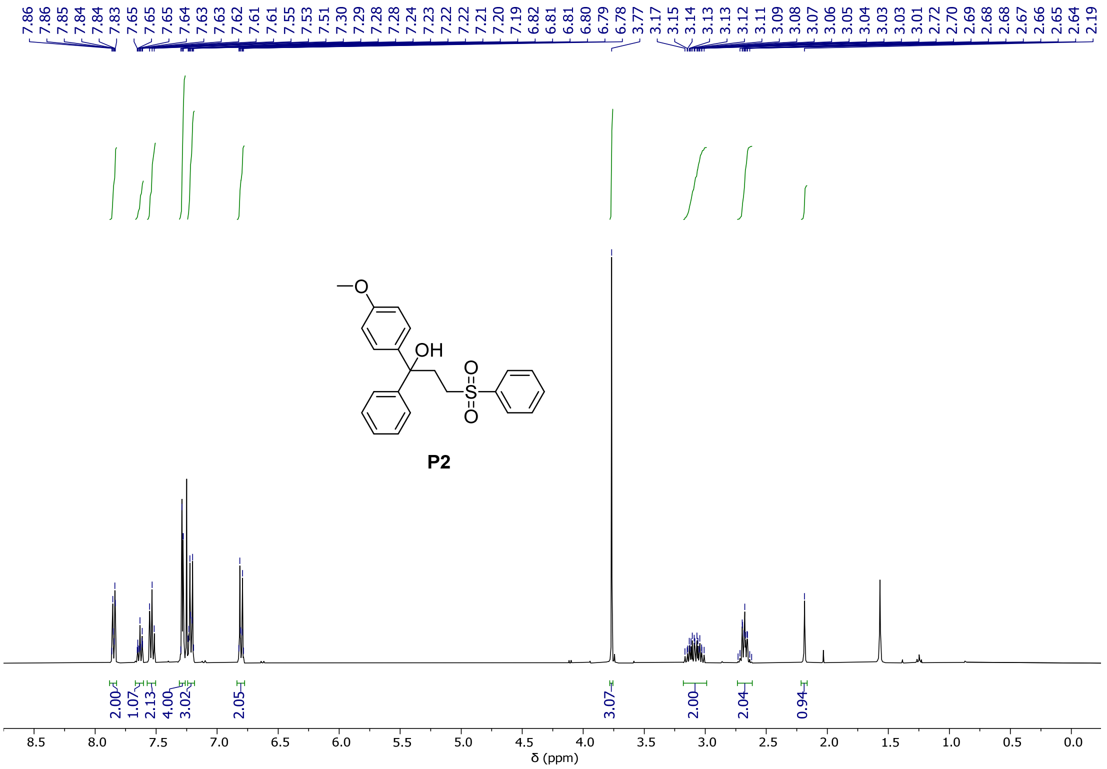

Fig. S18. ^{1}H NMR spectrum of **P2**, recorded in $CDCl_3$ at 400 MHz.

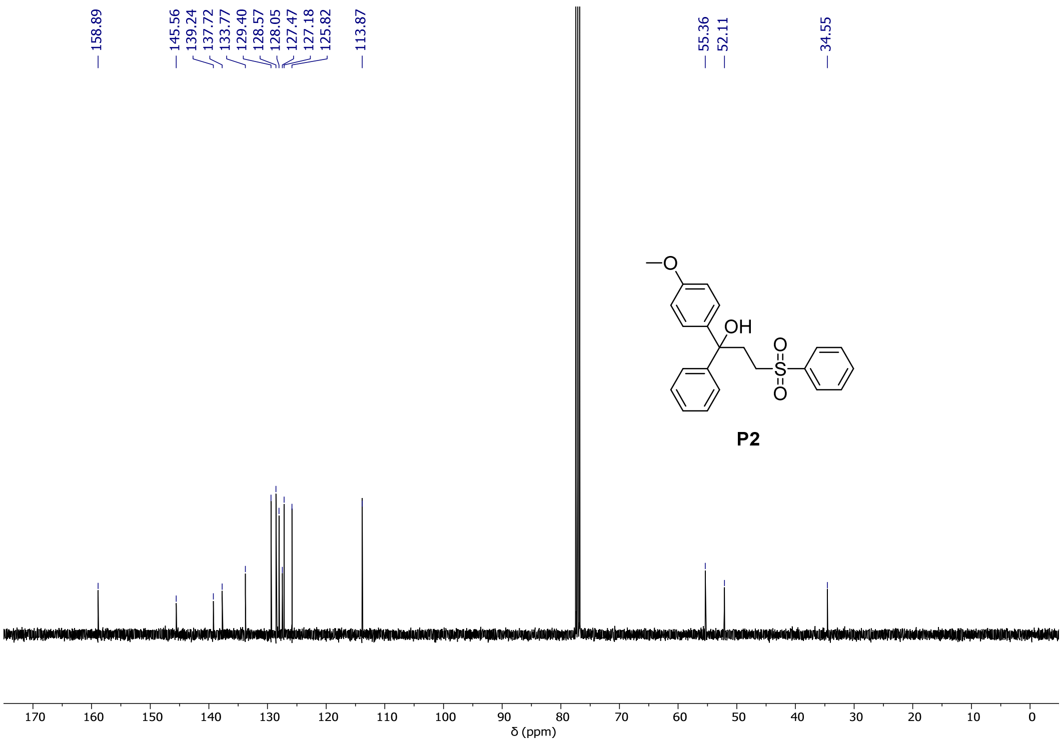

Fig. S19. $^{13}C\{^{1}H\}$ NMR spectrum of **P2**, recorded in $CDCl_3$ at 101 MHz.

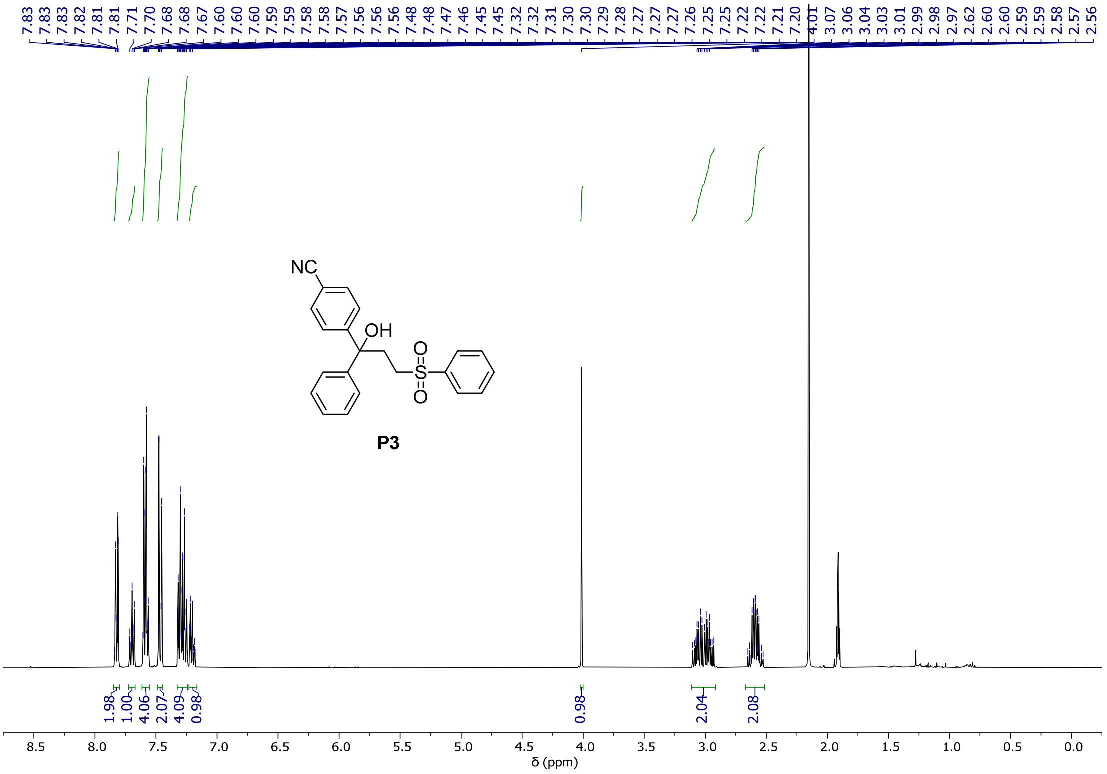

Fig. S20. ^{1}H NMR spectrum of **P3**, recorded in CD_3CN at 400 MHz.

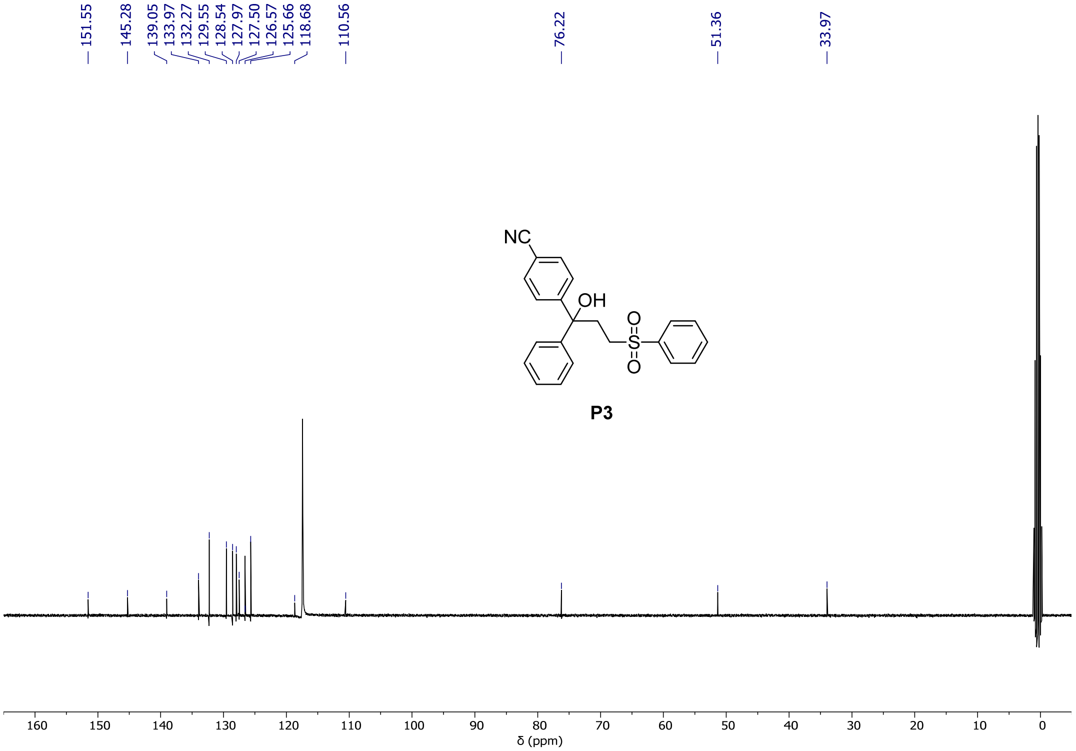

Fig. S21. $^{13}C\{^{1}H\}$ NMR spectrum of **P3**, recorded in CD_3CN at 101 MHz.

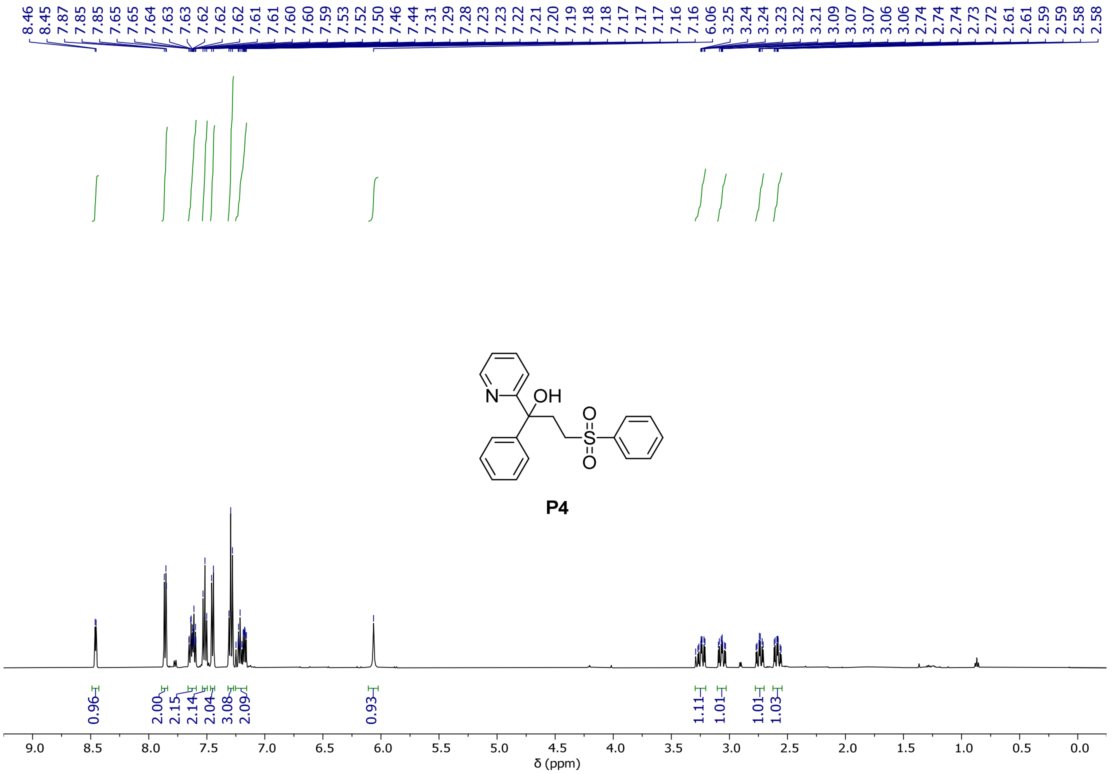

Fig. S22. ^{1}H NMR spectrum of **P4**, recorded in $CDCl_3$ at 500 MHz.

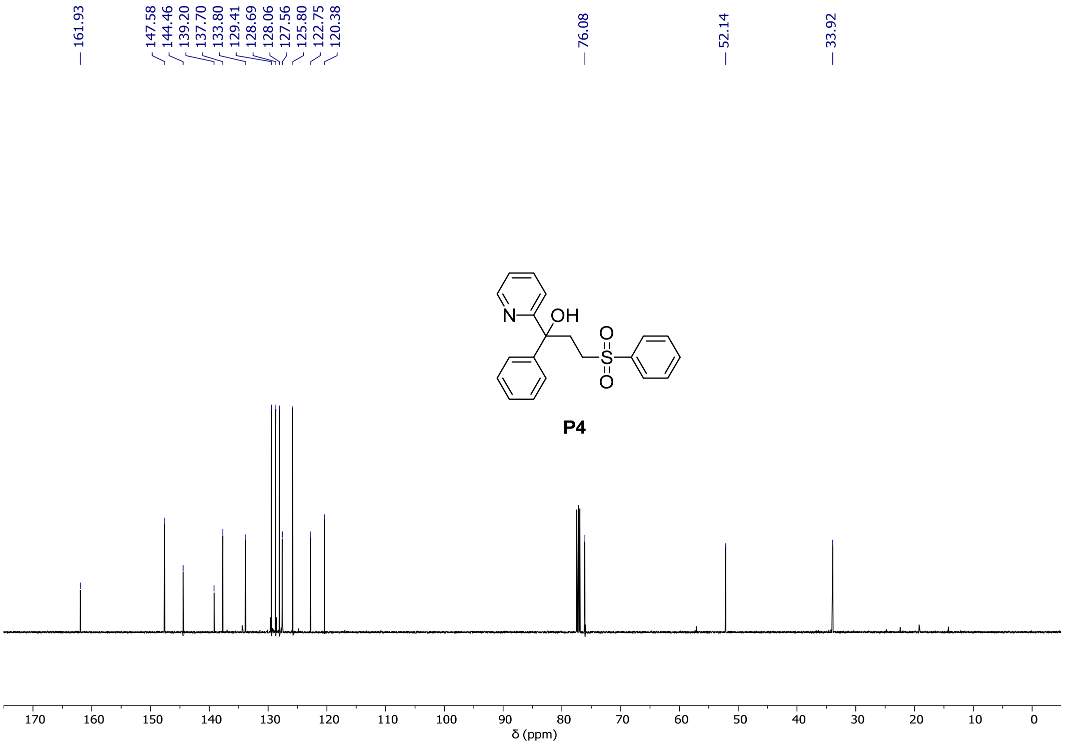

Fig. S23. $^{13}C\{^1H\}$ NMR spectrum of **P4**, recorded in $CDCl_3$ at 126 MHz.

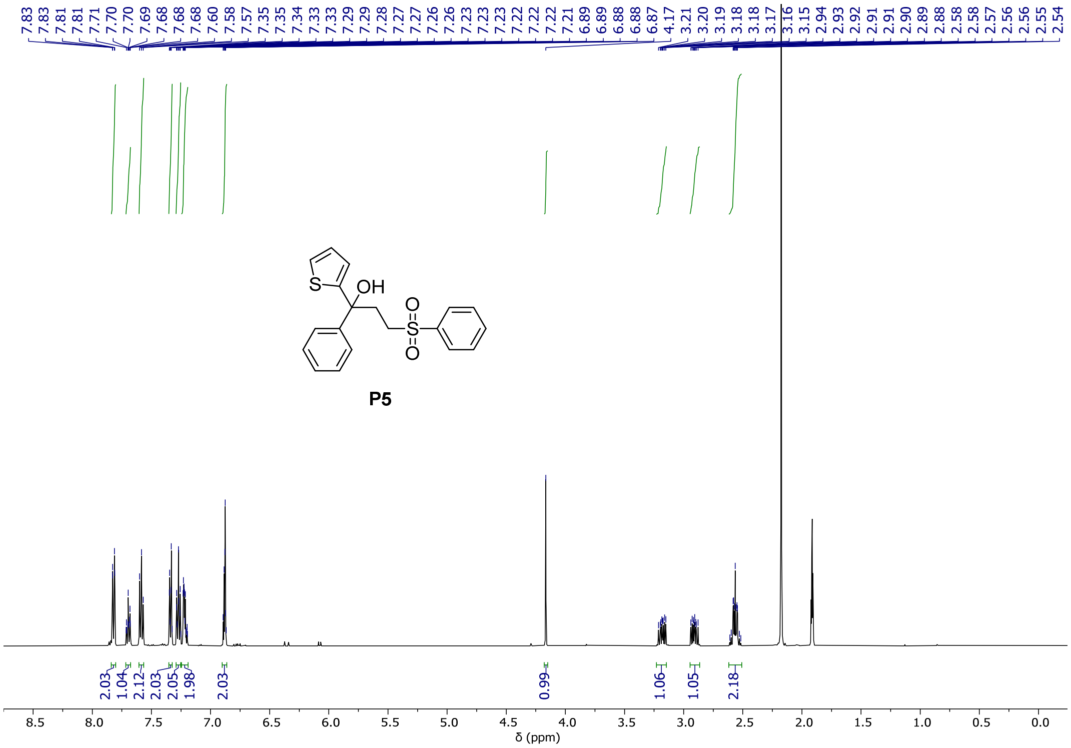

Fig. S24. ^{1}H NMR spectrum of **P5**, recorded in CD_3CN at 500 MHz.

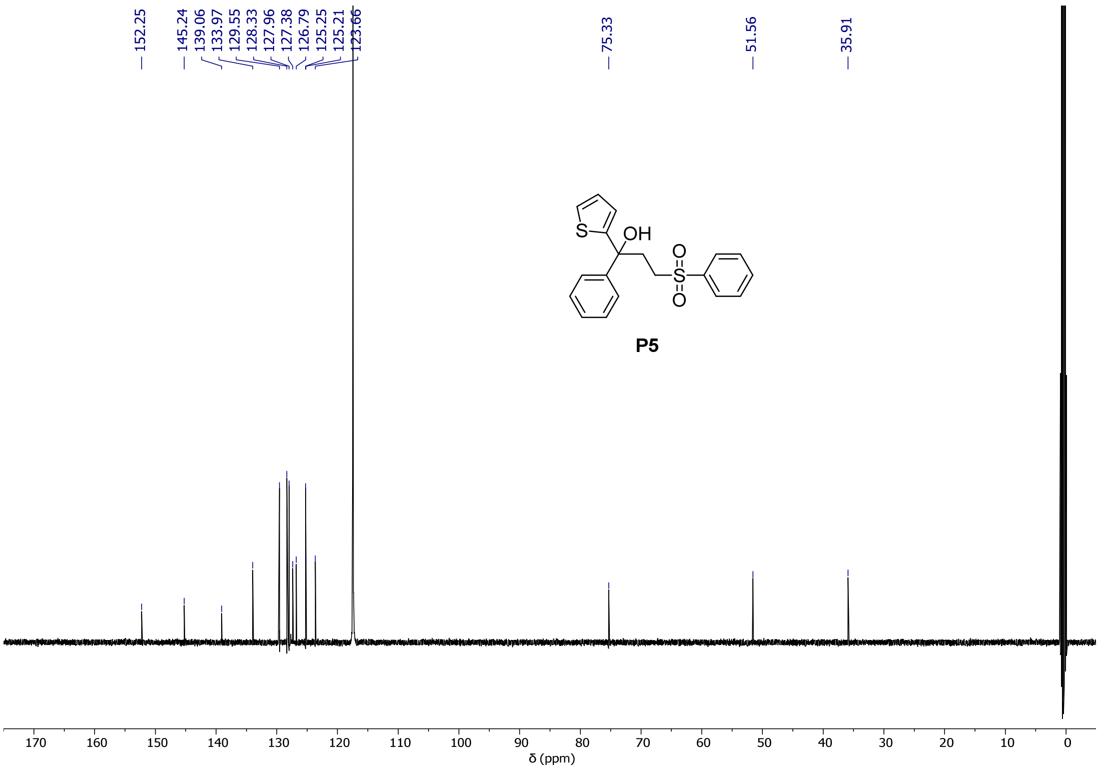

Fig. S25. ^{13}C{^{1}H} NMR spectrum of **P5**, recorded in CD_3CN at 126 MHz.

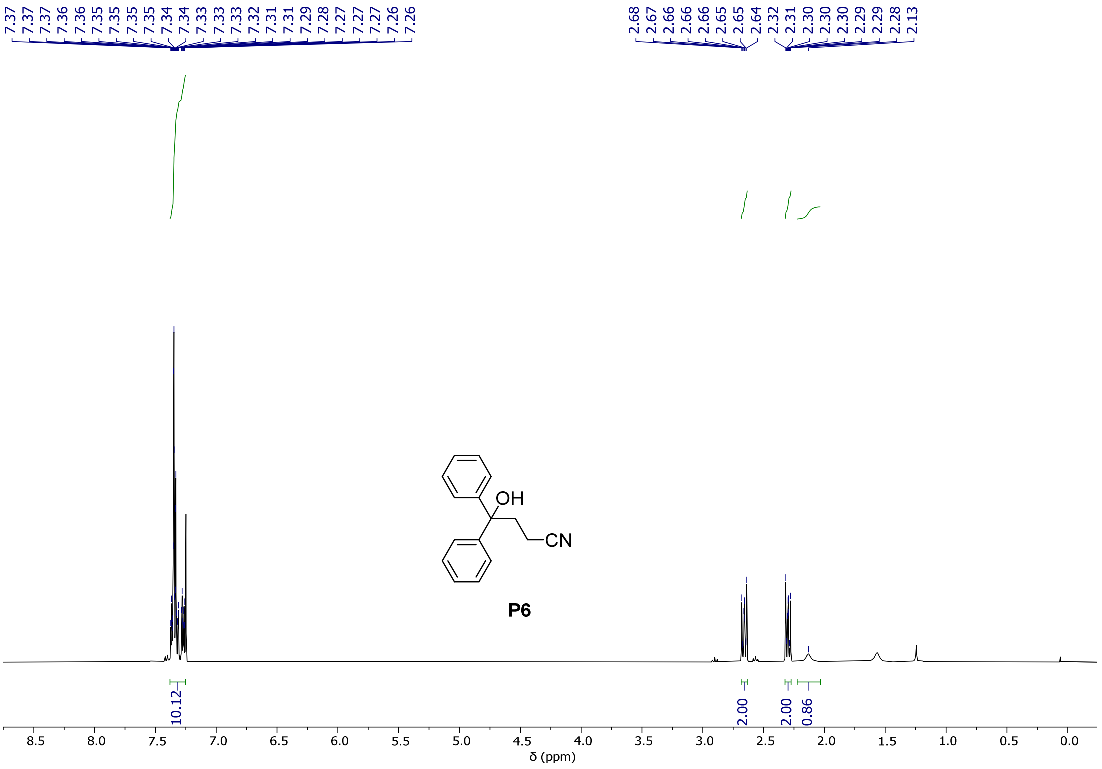

Fig. S26. ^1H NMR spectrum of **P6**, recorded in $CDCl_3$ at 400 MHz.

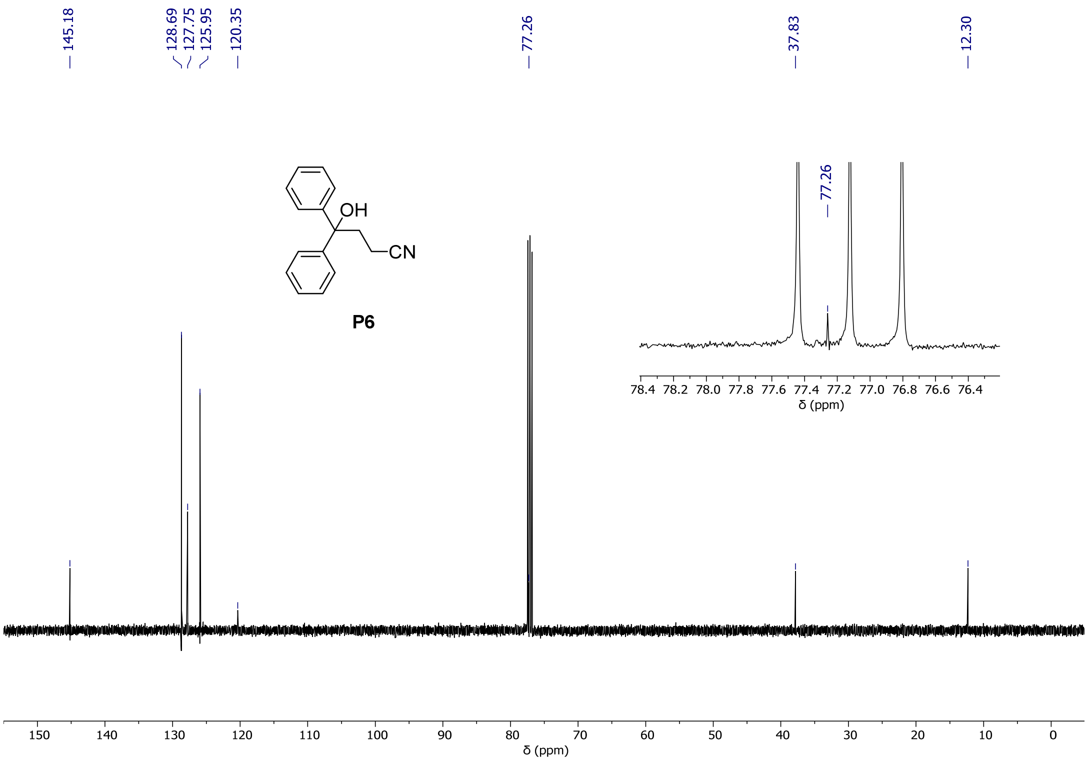

Fig. S27. $^{13}C\{^1H\}$ NMR spectrum of **P6**, recorded in $CDCl_3$ at 101 MHz.

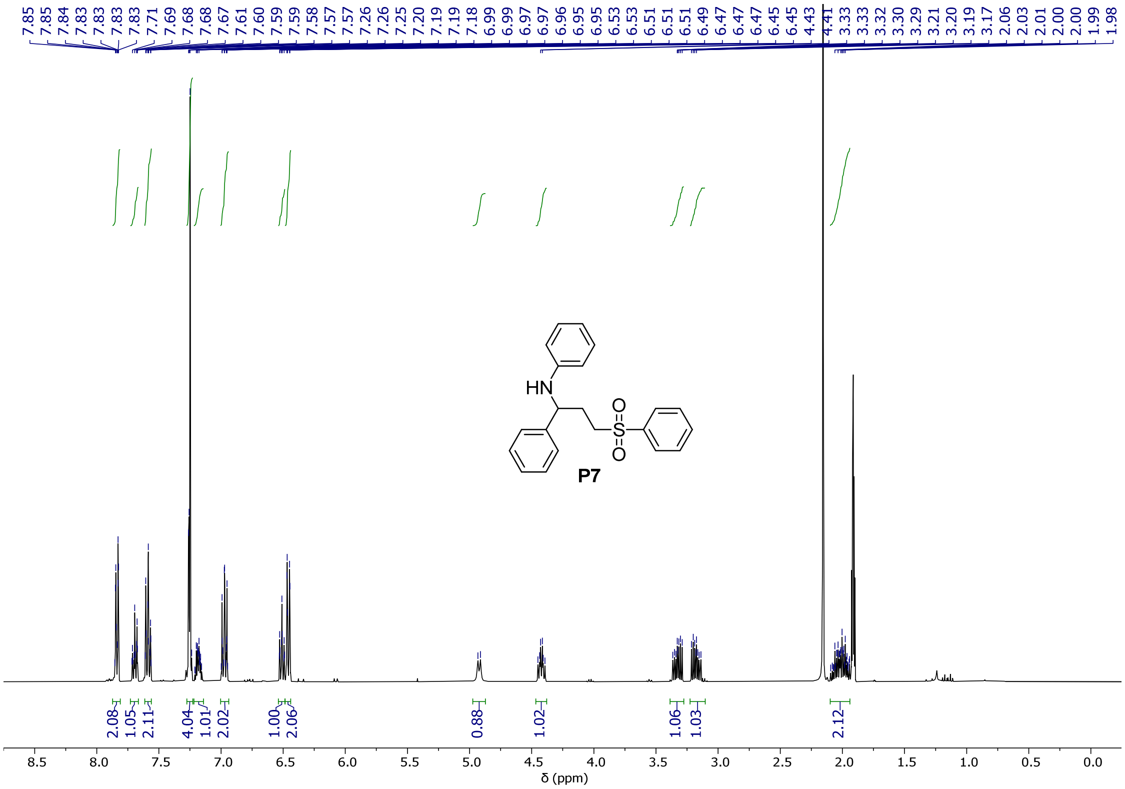

Fig. S28. ^{1}H NMR spectrum of **P7**, recorded in CD_3CN at 400 MHz.

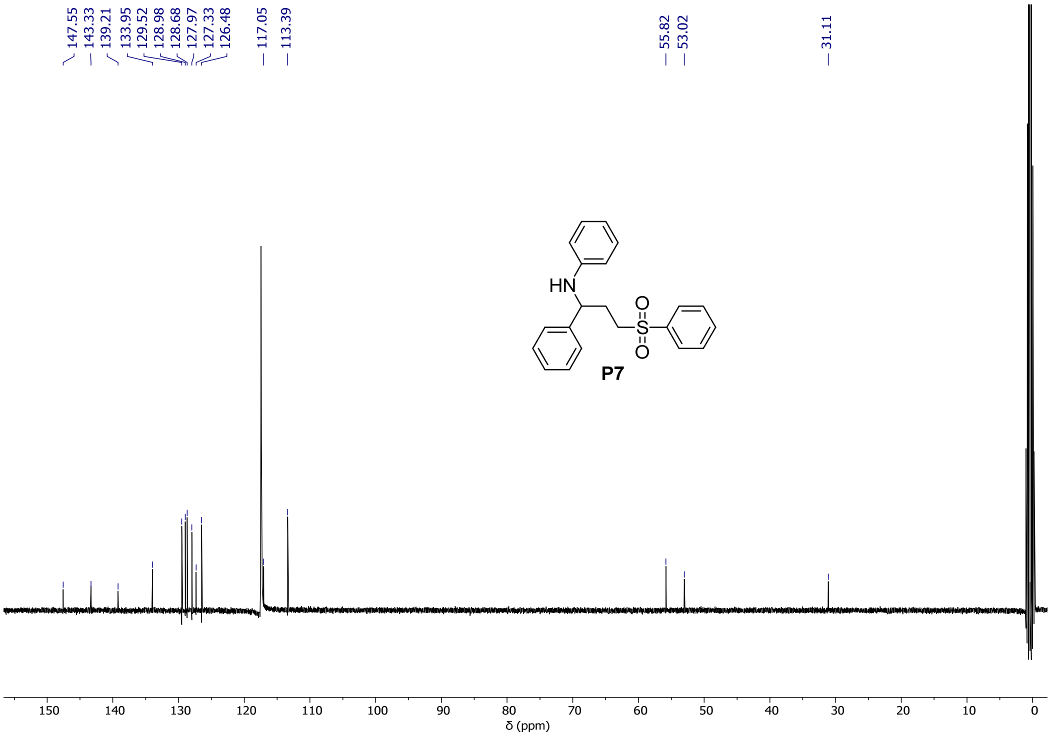

Fig. S29. $^{13}C\{^{1}H\}$ NMR spectrum of **P7**, recorded in CD_3CN at 101 MHz.

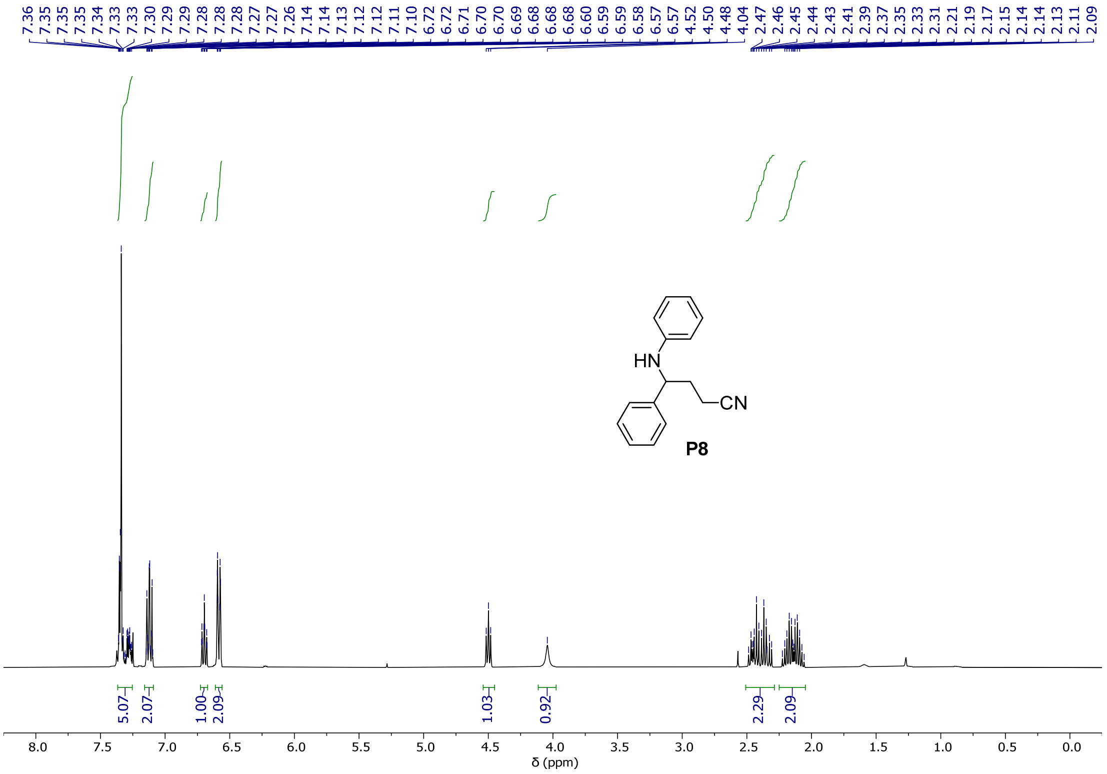

Fig. S30. ^{1}H NMR spectrum of **P8**, recorded in $CDCl_3$ at 400 MHz.

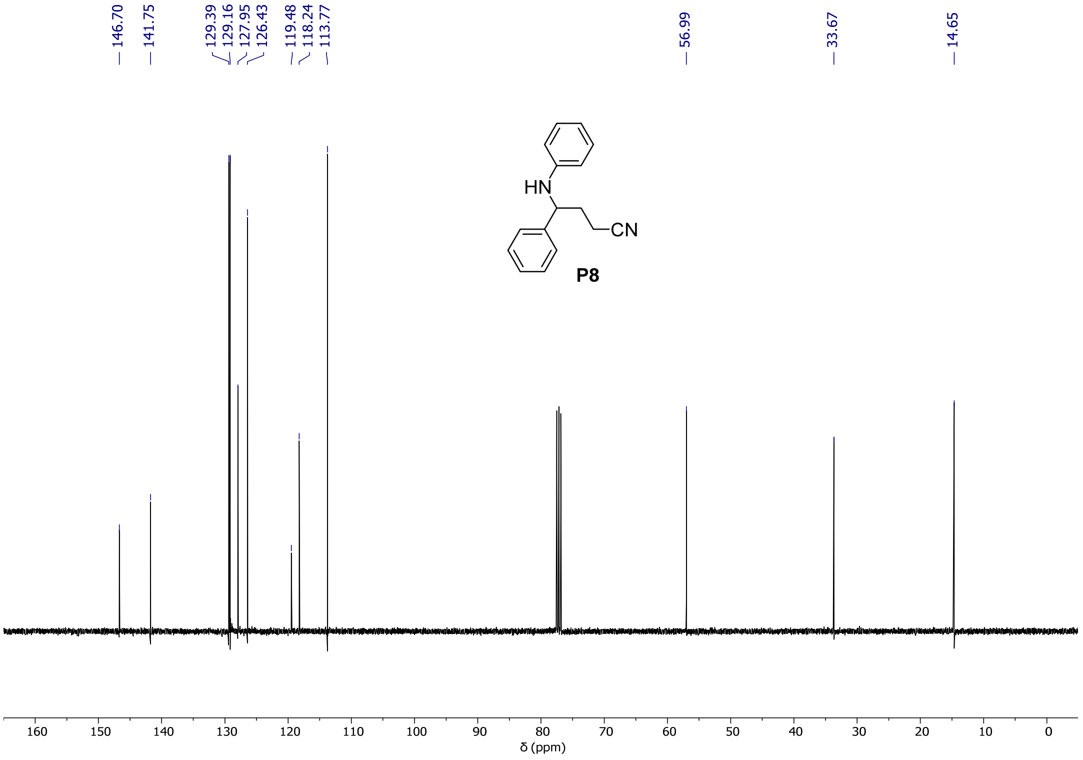

Fig. S31. $^{13}C\{^{1}H\}$ NMR spectrum of **P8**, recorded in $CDCl_3$ at 101 MHz.

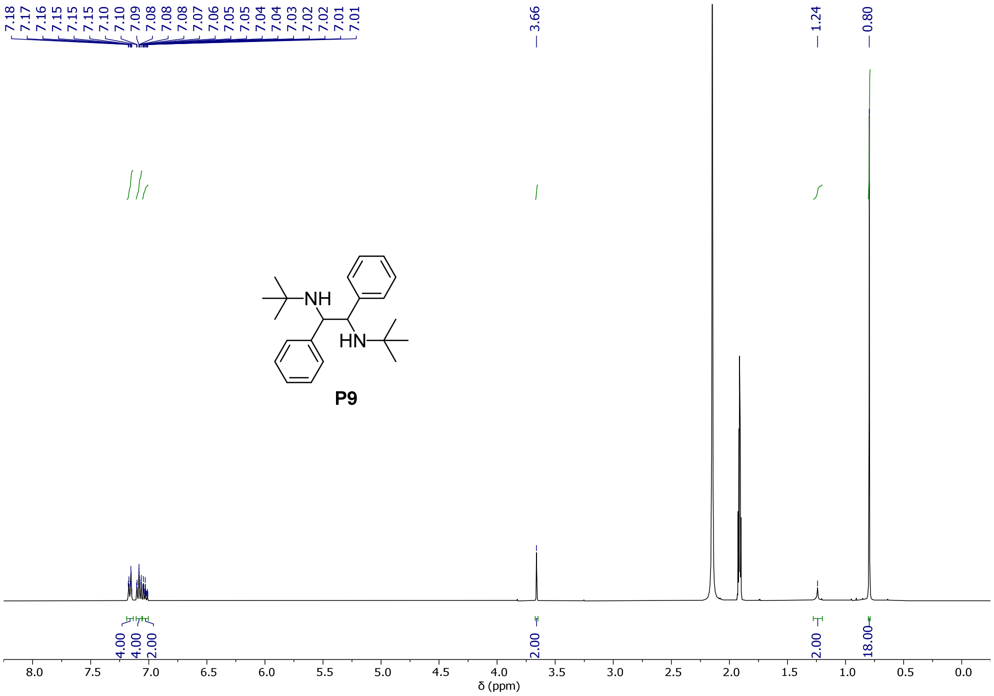

Fig. S32. ^{1}H NMR spectrum of **P9**, recorded in CD_3CN at 400 MHz.

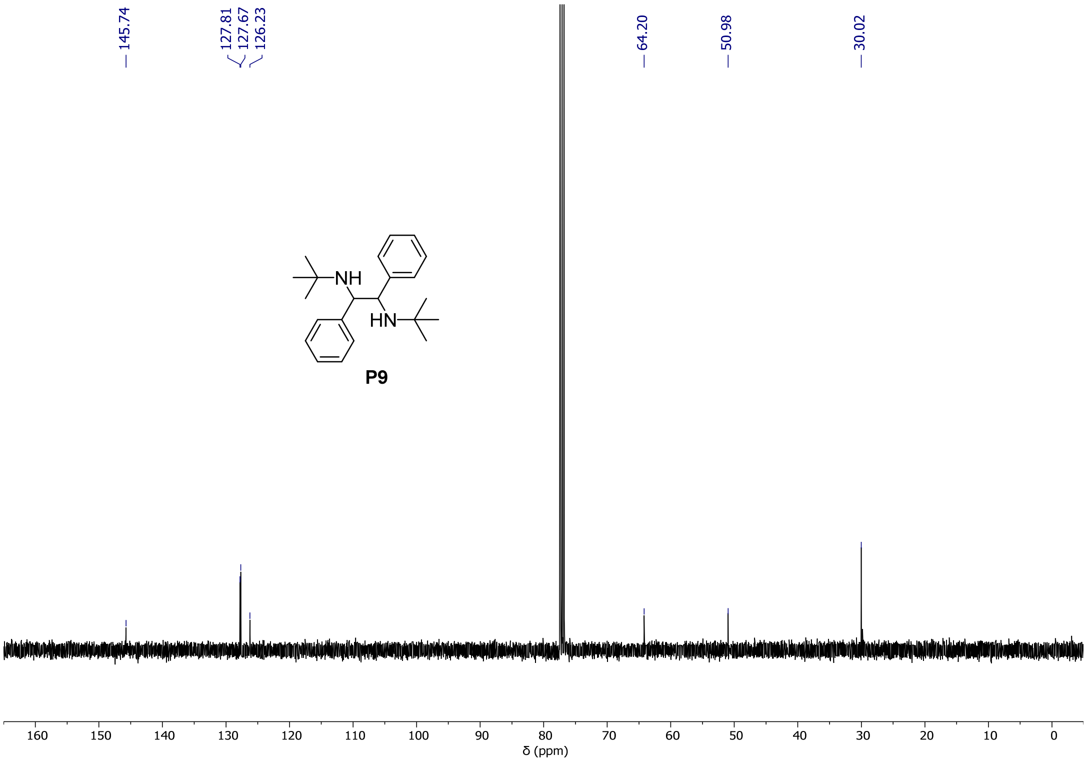

Fig. S33. $^{13}C\{^{1}H\}$ NMR spectrum of **P9**, recorded in $CDCl_3$ at 101 MHz.

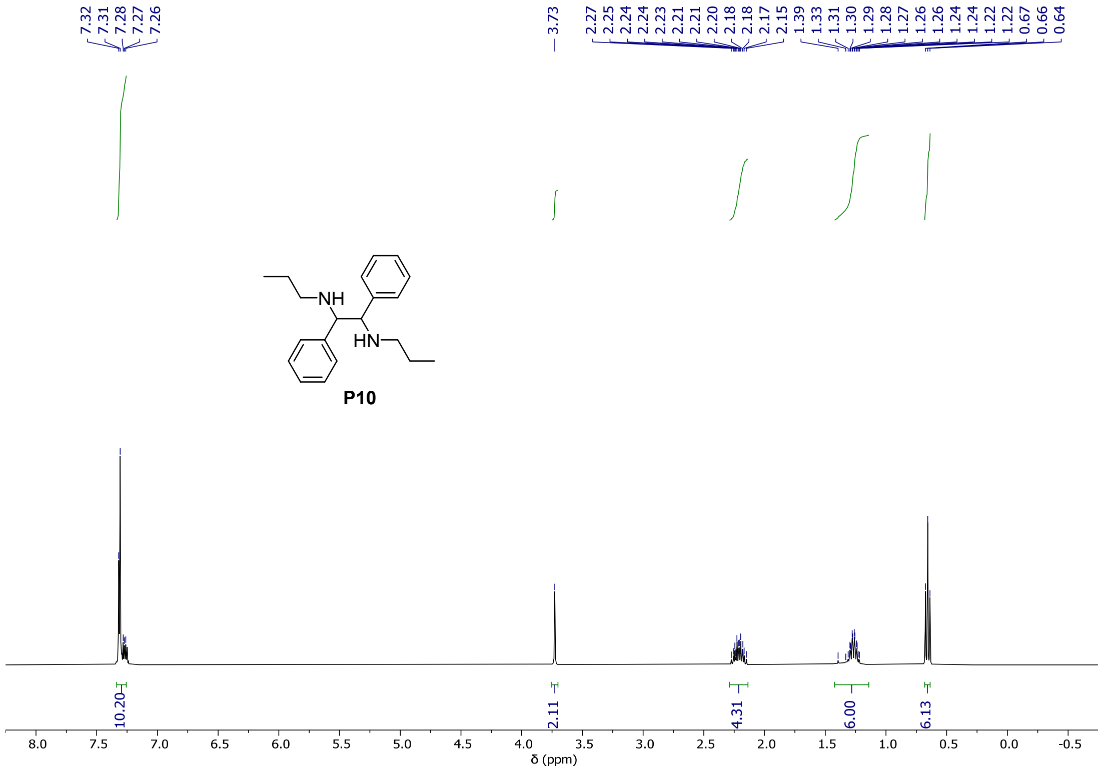

Fig. S34. ^{1}H NMR spectrum of **P10**, recorded in $CDCl_3$ at 400 MHz.

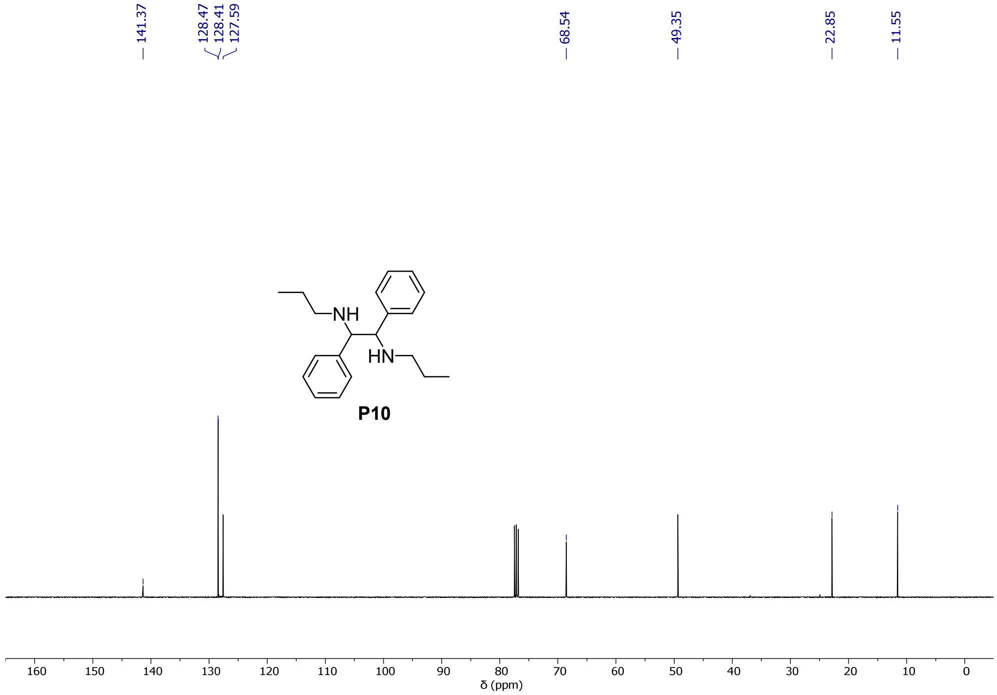

Fig. S35. ${}^{13}C\{{}^{1}H\}$ NMR spectrum of **P10**, recorded in $CDCl_3$ at 101 MHz.

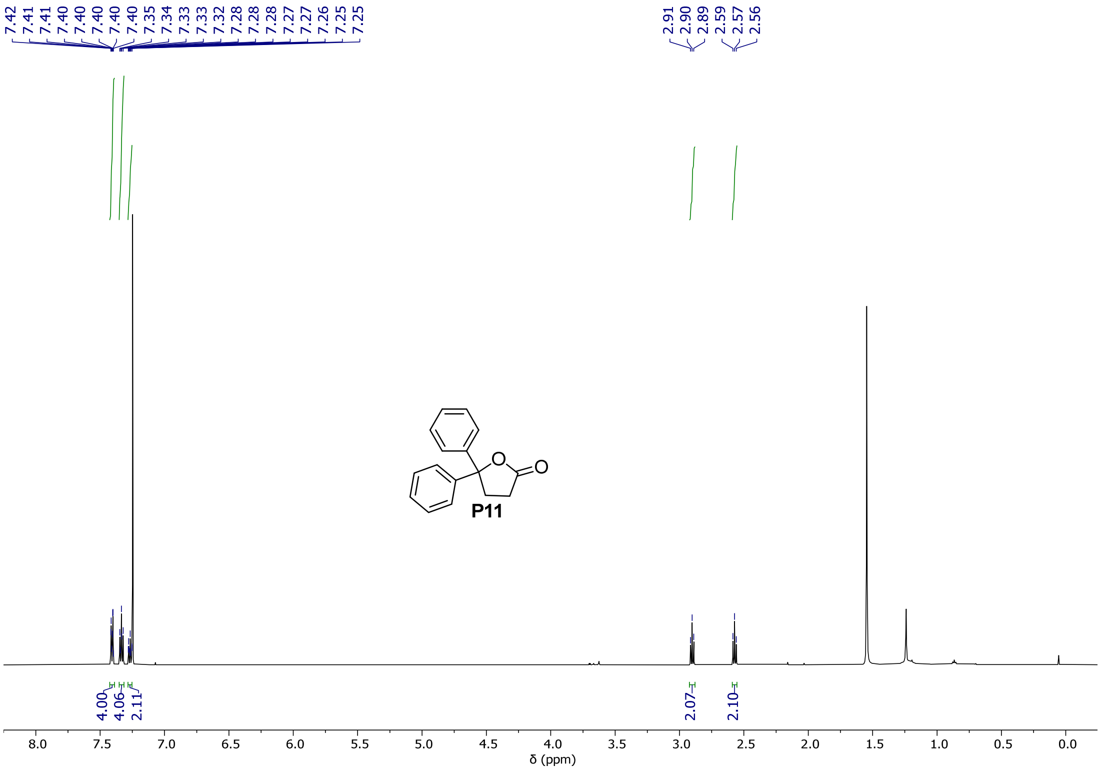

Fig. S36. ¹H NMR spectrum of **P11**, recorded in $CDCl_3$ at 600 MHz

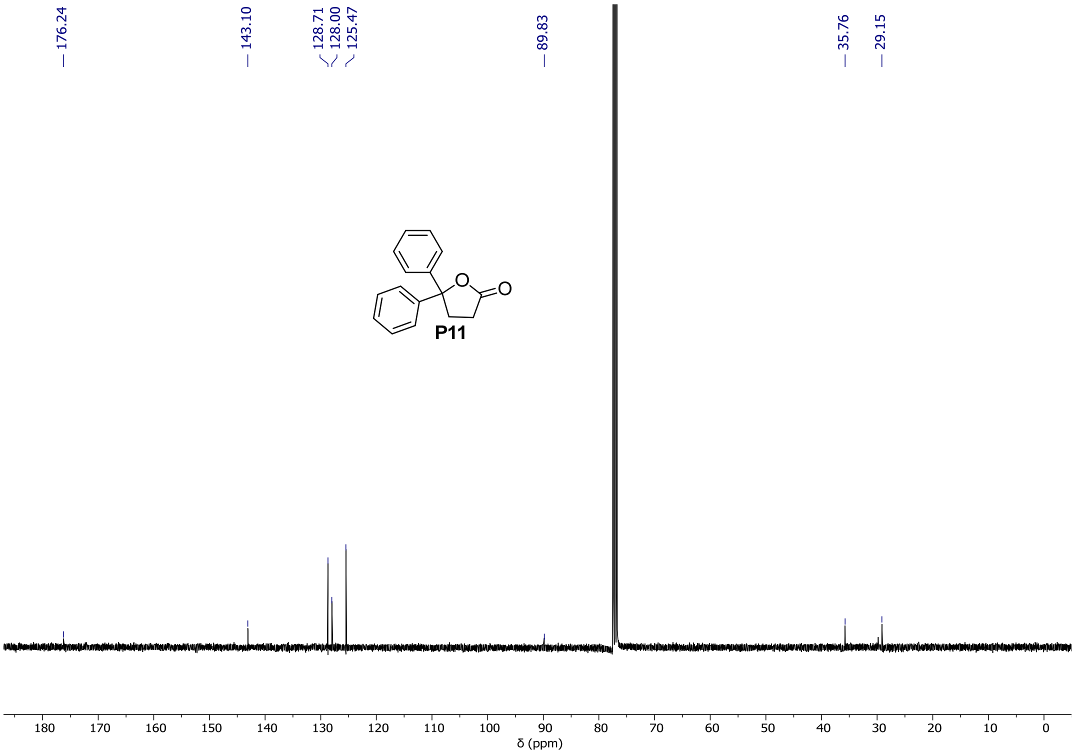

Fig. S37. $^{13}C\{^1H\}$ NMR spectrum of **P11**, recorded in $CDCl_3$ at 101 MHz.

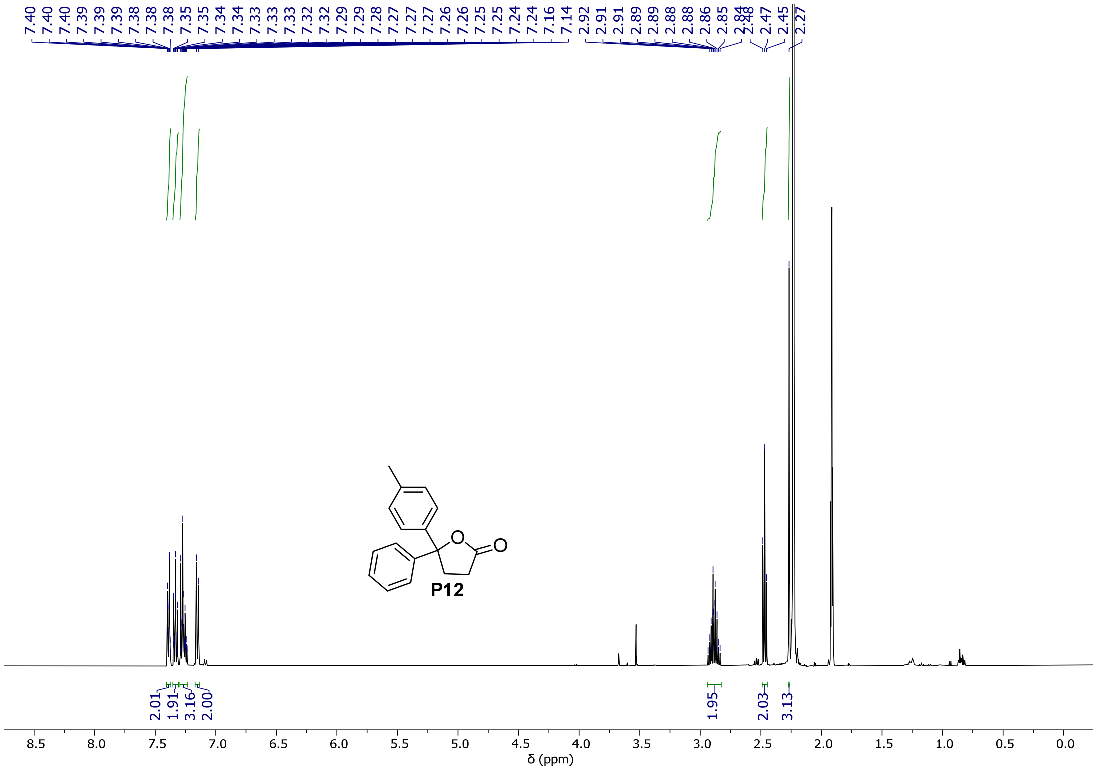

Fig. S38. ^{1}H NMR spectrum of **P12**, recorded in CD_3CN at 500 MHz.

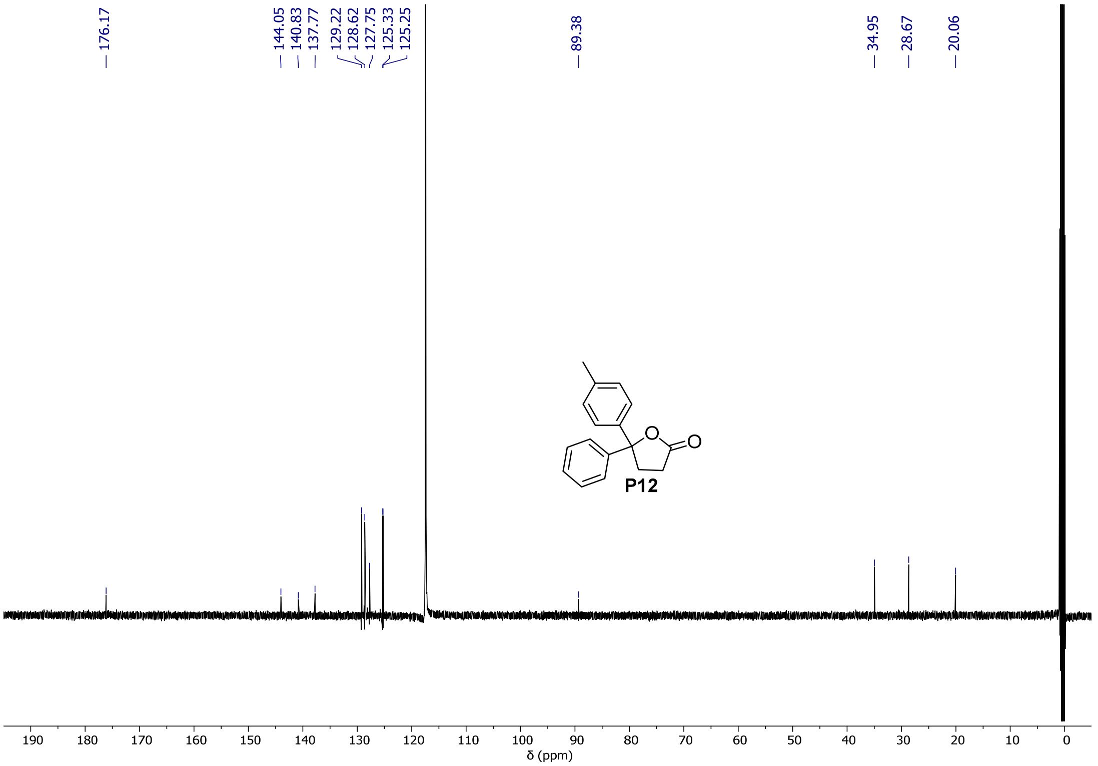

Fig. S39. $^{13}C\{^{1}H\}$ NMR spectrum of **P12**, recorded in CD_3CN at 126 MHz.

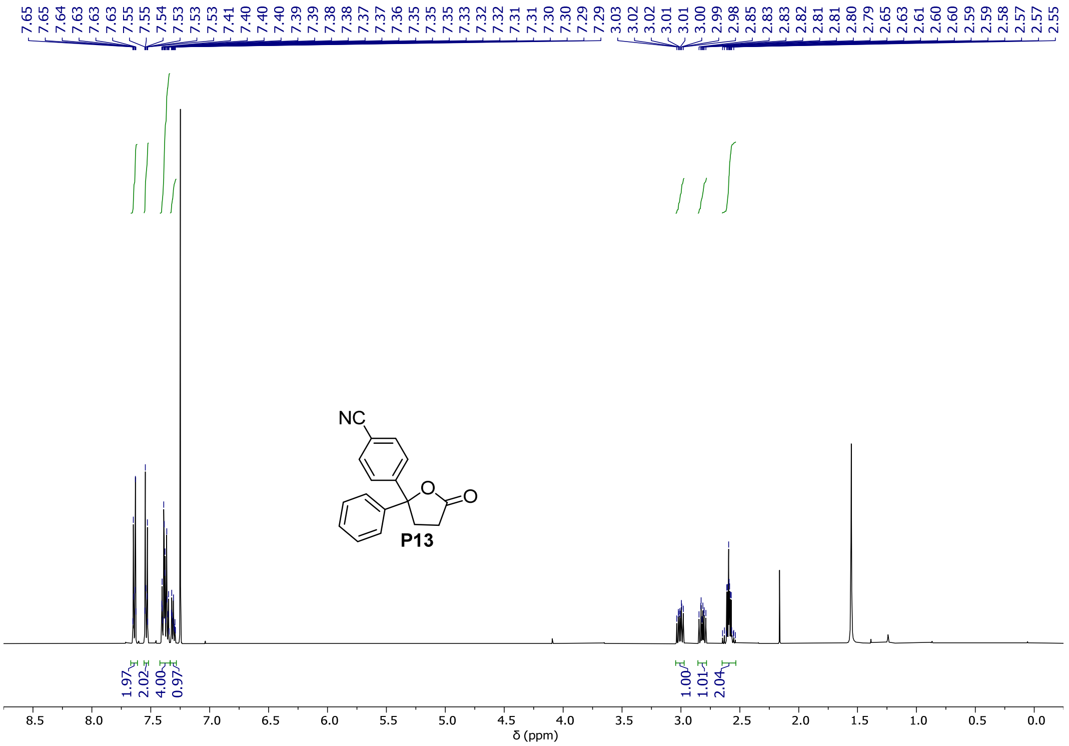

Fig. S40. ^{1}H NMR spectrum of **P13**, recorded in $CDCl_3$ at 500 MHz.

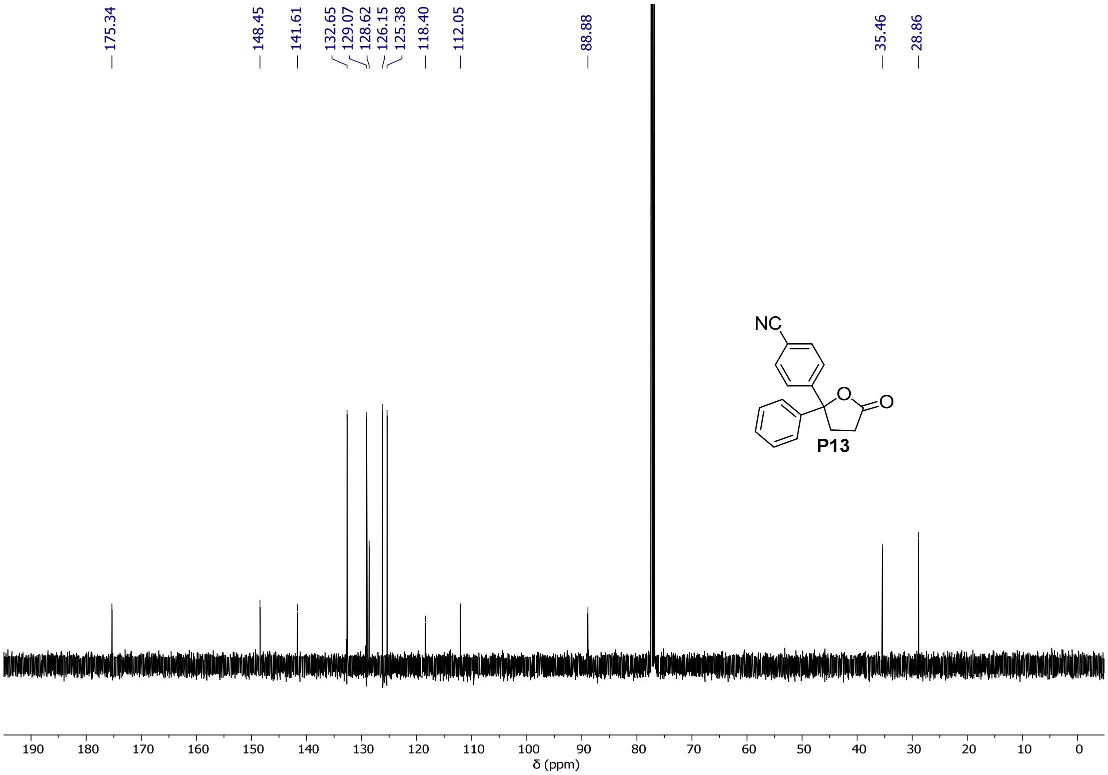

Fig. S41. $^{13}C\{^{1}H\}$ NMR spectrum of **P13**, recorded in $CDCl_3$ at 126 MHz.

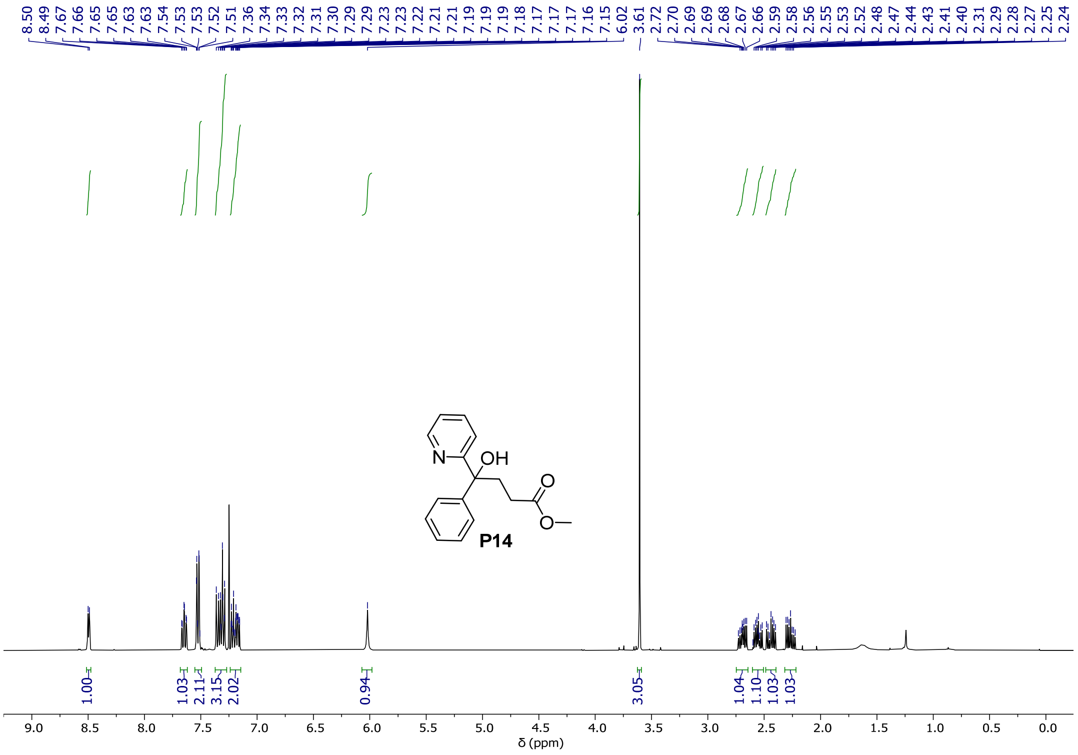

Fig. S42. ^{1}H NMR spectrum of **P14**, recorded in $CDCl_3$ at 400 MHz

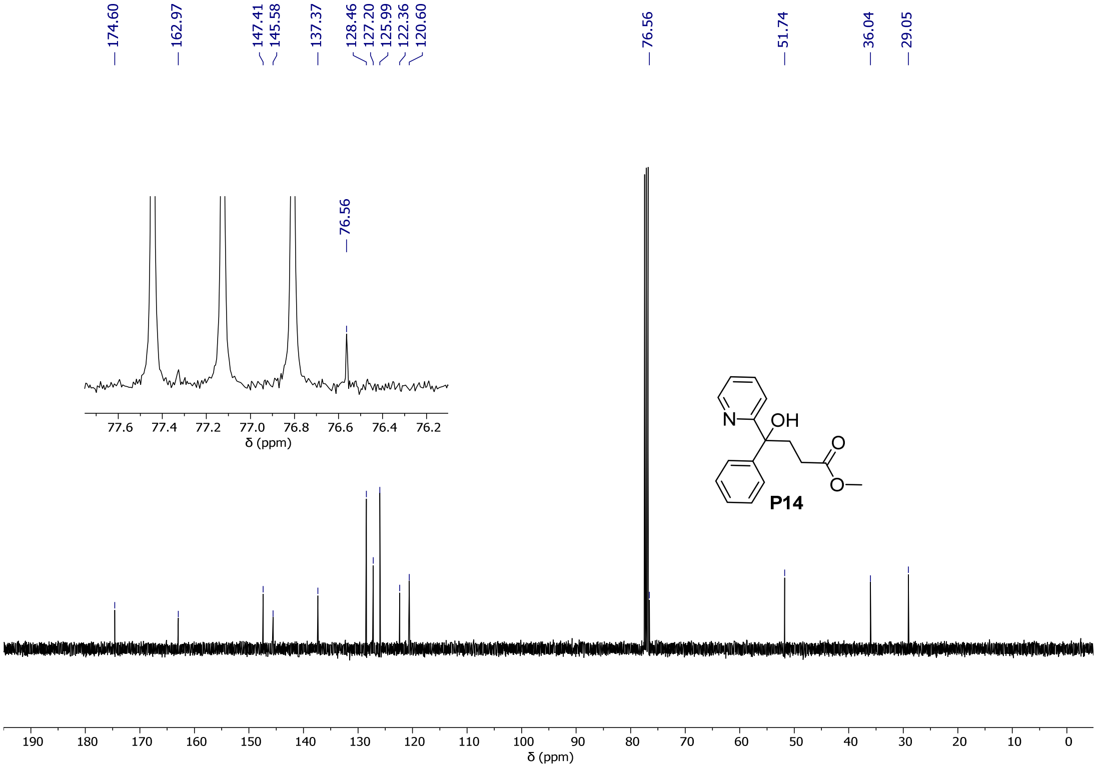

Fig. S43. ^{1}H NMR spectrum of **P14**, recorded in $CDCl_3$ at 101 MHz

References

1 J.-H. Shon and T. S. Teets, *Inorg. Chem.*, 2017, **56**, 15295–15303.
2 R. N. Sampaio, D. C. Grills, D. E. Polyansky, D. J. Szalda and E. Fujita, *J. Am. Chem. Soc.*, 2020, **142**, 2413–2428.
3 J.-H. Shon, S. Sittel and T. S. Teets, *ACS Catal.*, 2019, **9**, 8646–8658.
4 K. Dedeian, P. I. Djurovich, F. O. Garces, G. Carlson and R. J. Watts, *Inorg. Chem.*, 1991, **30**, 1685–1687.
